# Lumican deficiency dysregulates trophoblast function via PI3K/AKT and P53 signaling in preeclampsia

**DOI:** 10.1038/s41536-026-00493-8

**Published:** 2026-07-07

**Authors:** Xin Wang, Qi Zhang, Yi Ren, Kunzheng Fan, Chao Liu, Huijie Gao

**Affiliations:** College of Pharmacy, Jining Medical University, Jining, China

**Keywords:** Cell biology, Diseases, Molecular biology

## Abstract

Lumican, an extracellular matrix (ECM) component highly expressed in placenta, is implicated in preeclampsia (PE) pathogenesis, though its precise mechanisms in trophoblast function and PE development remain unclear. Transcriptomic analysis of a PE rat model induced by L-NAME revealed altered placental gene expression. Lumican expression progressively increased throughout rat and human gestation but was significantly downregulated in PE patient and model placentas. Functional assays showed shRNA-mediated *lumican* knockdown inhibited trophoblast proliferation, migration, and invasion while promoting apoptosis. This knockdown also influenced the phosphorylation of key proteins within the PI3K/AKT and P53 signaling pathways. Co-treatment with the PI3K agonist 740 Y-P activated this pathway, enhancing trophoblast proliferation and invasion, effects antagonized by *lumican* knockdown. In vivo, adenovirus-mediated *lumican* shRNA induced PE-like phenotypes: elevated systolic blood pressure, increased 24 h urinary protein, higher plasma sFlt-1 levels and sFlt-1/PlGF ratio. Histopathological analyses demonstrated abnormal placental villi development and renal glomerular endothelial damage. Immunohistochemistry confirmed lumican deficiency reduced PI3K, AKT, and MDM2 while upregulating P53 in placental tissues. Collectively, our findings demonstrate that reduced lumican expression contributes to PE by impairing trophoblast function via disruption of the PI3K/AKT and P53 signaling pathway, thus providing a mechanistic basis for its further exploration as a diagnostic marker and therapeutic target.

**Figure:**
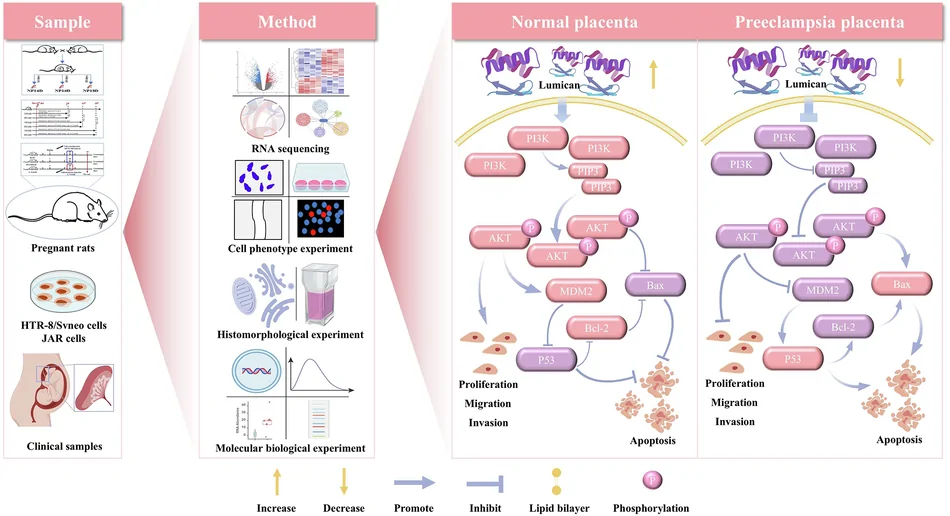

## Introduction

The emergence of gestational hypertension is a characteristic of preeclampsia (PE), a severe pregnancy-related illness that usually manifests after the 20th week of pregnancy^1–4^. As a complex multisystem disorder, PE places mothers at considerable risk for severe side effects such as HELLP syndrome, cerebrovascular incidents, and multiorgan failure involving hepatic and renal systems^5^. These maternal and fetal risks highlight the pressing need to clarify the fundamental mechanisms of PE to facilitate early diagnosis and targeted therapeutic strategies aimed at minimizing morbidity and mortality.

The placenta is widely recognized as the origin of the development of PE, as symptoms typically resolve after delivery^6^. Syncytiotrophoblast cells (STBs) and extravillous trophoblast cells (EVTs) differentiated from cytotrophoblast cells serve as central mediators in placental function and are critically involved in the pathogenesis of PE. According to the most recent two-stage model, STB stress is a shared feature in both preterm and term PE^7–9^. Stressed STB exhibit abnormal secretion of pro-inflammatory cytokines, reactive oxygen species, and anti-angiogenic factors-such as soluble fms-like tyrosine kinase-1 (sFlt1)—into the maternal circulation^10^. These factors contribute to systemic endothelial dysfunction and multi-organ impairment, thereby driving the clinical manifestations of PE. However, the underlying causes of STB stress differ between subtypes: impaired spiral artery remodeling is widely recognized as the primary contributor to preterm PE, whereas the initiating mechanisms in term PE remain incompletely understood and require further investigation.

A key determinant of trophoblast function, which may underlie these stress responses and functional deficits, is their dynamic crosstalk with the surrounding extracellular matrix (ECM).Trophoblast interactions with the ECM are vital for regulating cell proliferation, differentiation, and invasive potential. Among ECM components, Small Leucine-Rich Proteoglycans (SLRPs) have been known for their vital role in placental development. These SLRPs significantly affect trophoblast proliferation, migration, and invasion, and their dysregulation has been associated with the onset and progression of PE^11^.

Lumican, a member of the class II SLRPs, is encoded by a gene located on the distal end of human chromosome 12. This gene covers approximately 6.9 kb and consists of three exons separated by two introns^12,13^. Structurally, lumican is a 38-kDa core protein comprising four specific regions: (1) a 16-amino acid signal sequence, (2) an acidic N-terminal region containing a sulfated tyrosine and a disulfide linkage, (3) a central domain enriched with 6 to 10 leucine-rich repeat (LRR) motifs, and (4) a C-terminal region harboring two conserved cysteine residues^14^. Lumican shows a broad tissue expression profile, mainly in the skin, intervertebral discs, placenta, lungs, liver, and skeletal muscles. Recently, growing interest has been directed toward lumican’s role in regulating tumor cell dynamics^15–19^. Interestingly, trophoblasts, due to their invasive potential resembling epithelial-mesenchymal transition (EMT) observed in malignancies, are often called “cancer-like” cells^20^. Although previous research has indicated lumican’s involvement in PE pathophysiology by modulating HTR-8/SVneo trophoblast cell behavior, the specific molecular mechanisms and pathways through which lumican has its regulatory effects remain to be fully elucidated^21^.

The P53 and PI3K/AKT signaling pathways regulate trophoblast function. Recent findings suggest that recombinant Syntaxin-2 may play a therapeutic role in PE by PI3K/AKT pathway activation, promoting trophoblast invasion, migration, and proliferation^22^. Supporting this, Liu et al. demonstrated that Protein O-Fucosyltransferase 1 promotes trophoblast growth by stimulating the PI3K/AKT pathway. Simultaneously, trophoblast function is strongly affected by the P53 signaling axis^23^. A decrease in protein disulfide isomerase A3 (PDIA3) expression leads to the activation of the murine double minute2 (MDM2, negative regulator of P53)/p53/p21 pathway, triggering trophoblast apoptosis and inhibiting proliferation^24^. The protective role of P53 inhibition against hydrogen peroxide-induced apoptosis in placental trophoblasts further emphasizes its significant involvement in cell survival mechanisms^25^. Previous experimental findings demonstrated that lumican expression is significantly associated with P53 regulation in trophoblasts. Reduced lumican levels markedly suppressed trophoblast cell proliferation while elevating P53 expression, indicating a potential regulatory association between lumican and the P53 signaling pathway in trophoblast function^21^.

Taken together, these findings indicate that lumican may regulate trophoblast function through the PI3K/AKT and P53 signaling pathways. Nevertheless, the exact molecular mechanisms remain unclear. To address this, the present study was designed to systematically examine how lumican affects trophoblast behavior and participates in PE pathogenesis. We integrated clinical placental analysis, functional experiments in trophoblast cell lines, and in vivo verification in a PE rat model to clarify the involved signaling mechanisms. This work primarily aims to provide mechanistic insight into the role of lumican in PE, which could support its future exploration as a diagnostic marker or therapeutic target.

## Results

### Upregulation of lumican during placental development

**Fig. 1:**
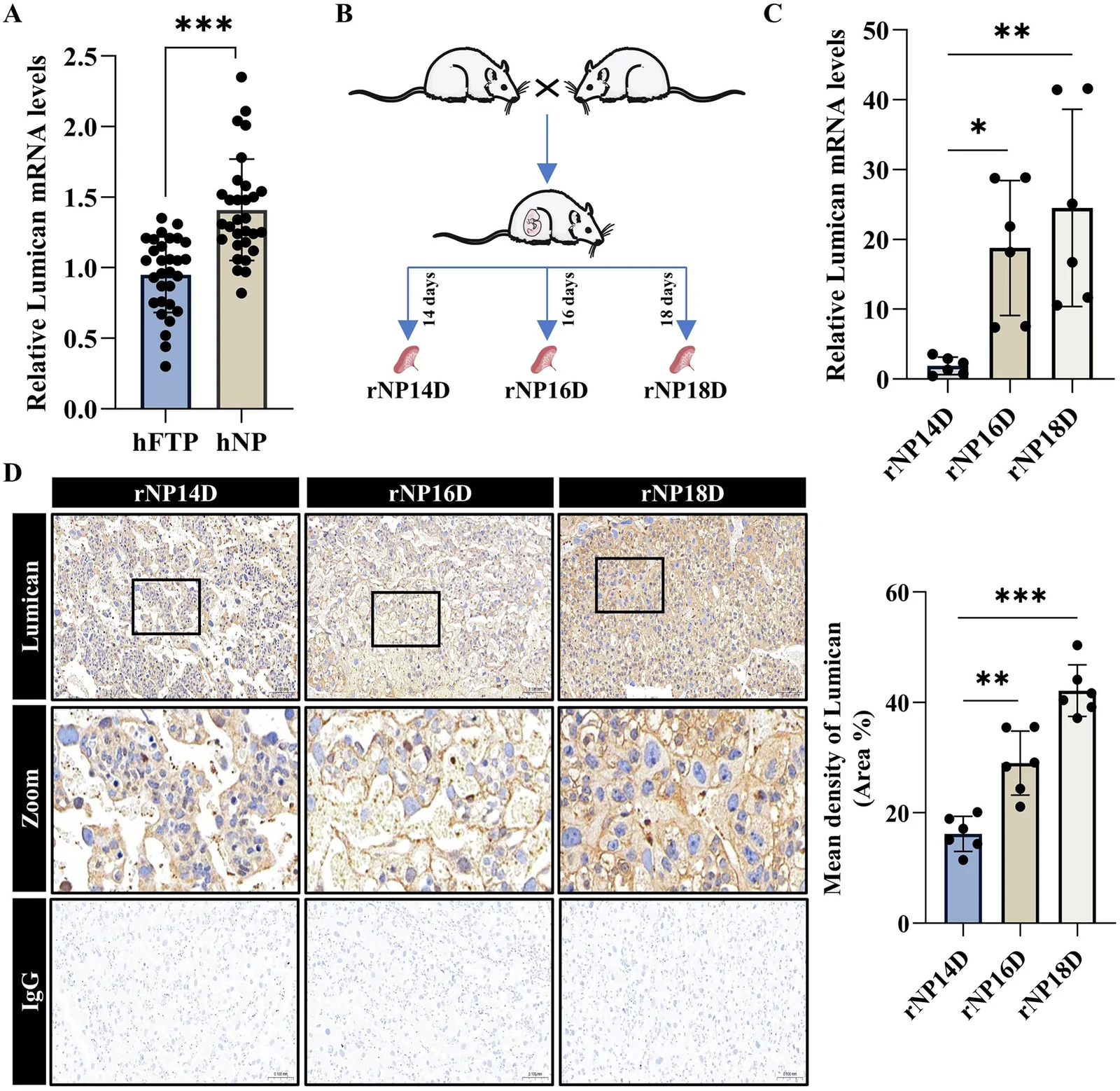
Upregulation of lumican during placental development. **A** Expression level of *lumican* mRNA in early-gestation chorionic tissues (first-trimester placenta, hFTP, *n* = 30) and term placentas from normal pregnant women (hNP, *n* = 30); **B** Establishment of rat placental development model. rNP14D: Rat at Gestational Day 14; rNP16D: Rat at Gestational Day 16; rNP18D: Rat at Gestational Day 18. **C** Expression level of *lumican* mRNA in rat placenta. **D** Expression level of lumican protein in rat placenta. The scale bar is 200 μm. *n* = 6. Data are presented as the mean ± SEM. Statistical significance was determined by unpaired t-test or one-way ANOVA with Tukey’s post hoc test. *P < 0.05, **P < 0.01, ***P < 0.001.

RT-qPCR analysis revealed a higher expression of *lumican* mRNA in the human normal term placenta (hNP) compared to the first-trimester placenta (hFTP) (Fig. 1A). Both RT-qPCR and immunohistochemical staining demonstrated that the level of lumican expression gradually increased in the placental tissues of rats (rNP) on the 14th, 16th, and 18th day of pregnancy (Fig. 1B–D).

### Significant changes in the PI3K/AKT and P53 signaling pathways during rat placental development

**Fig. 2:**
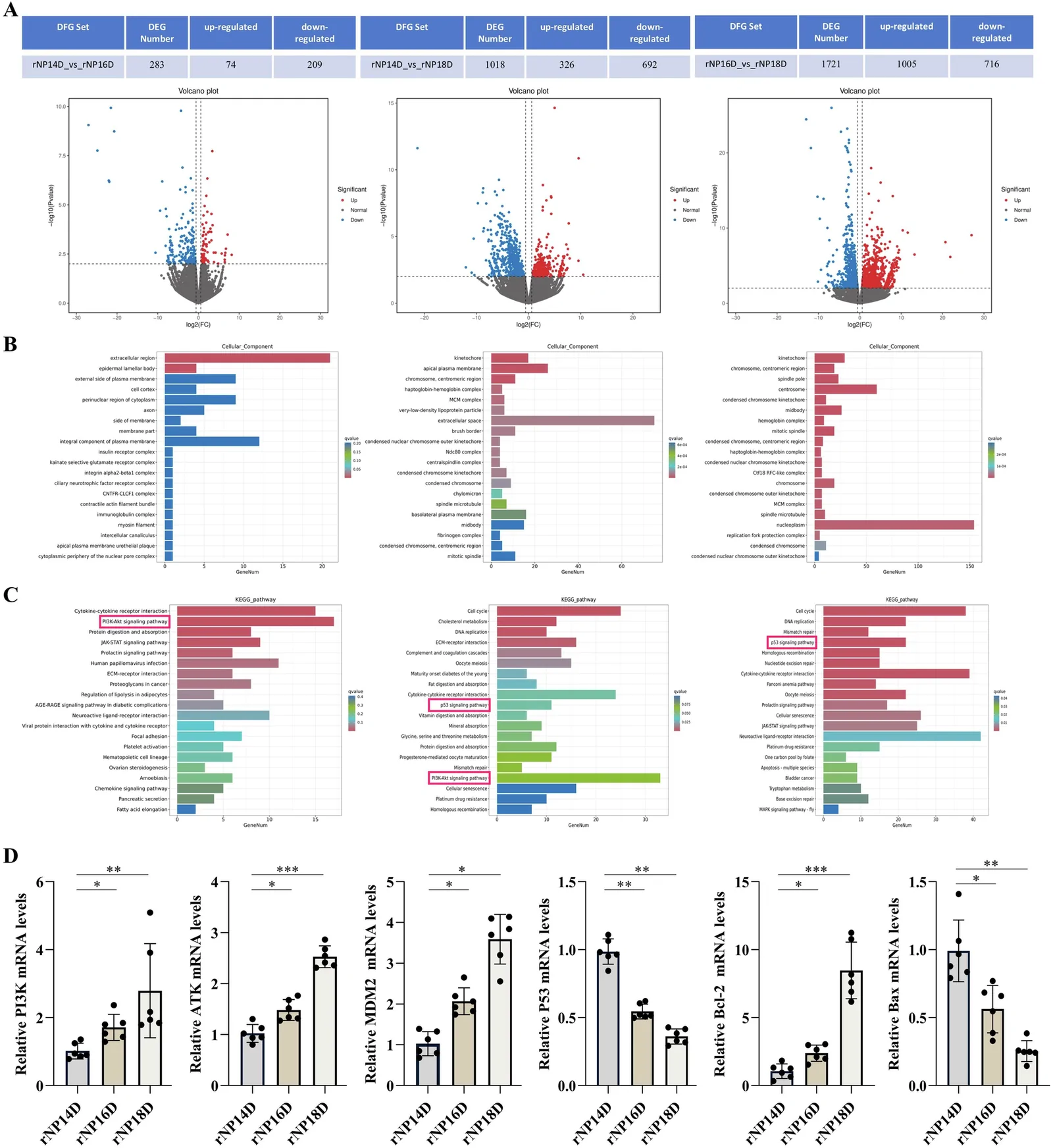
Significant changes in the PI3K/AKT and P53 signaling pathways during rat placental development. **A** Volcano Plot; **B** GO Enrichment; **C** KEGG Enrichment. **D** Expression level of *PI3K* mRNA, *AKT* mRNA, *MDM2* mRNA, *Bcl-2* mRNA, and *Bax* mRNA. *n* = 6. Data are presented as the mean ± SEM. Statistical significance was determined by one-way ANOVA with Tukey’s post hoc test for multiple comparisons. *P < 0.05, **P < 0.01.

Transcriptome analysis of rat placental tissue revealed dynamic gene expression changes across gestational stages. A total of 283 differentially expressed genes were identified between gestational days 14 and 16, and 1,018 genes between days 14 and 18. When comparing days 18 and 16, 1,721 differentially expressed genes were observed (Figs. 2A and S1A). Gene Ontology (GO) (Figs. 2B and S1B) and Kyoto Encyclopedia of Genes and Genomes (KEGG) enrichment analyses indicated that the P53 and PI3K/AKT signaling pathways exhibited significant alterations during placental development (Fig. 2C). There was an upregulation of the anti-apoptotic gene *Bcl-2* and a significant downregulation of the pro-apoptotic genes *Bax* and *P53*. In parallel, expression of *PI3K*, *AKT*, and *MDM2* mRNA was up-regulated (Fig. 2D), suggesting progressive activation of anti-apoptotic signaling with advancing gestation.

### Downregulation of lumican in PE placenta

**Fig. 3:**
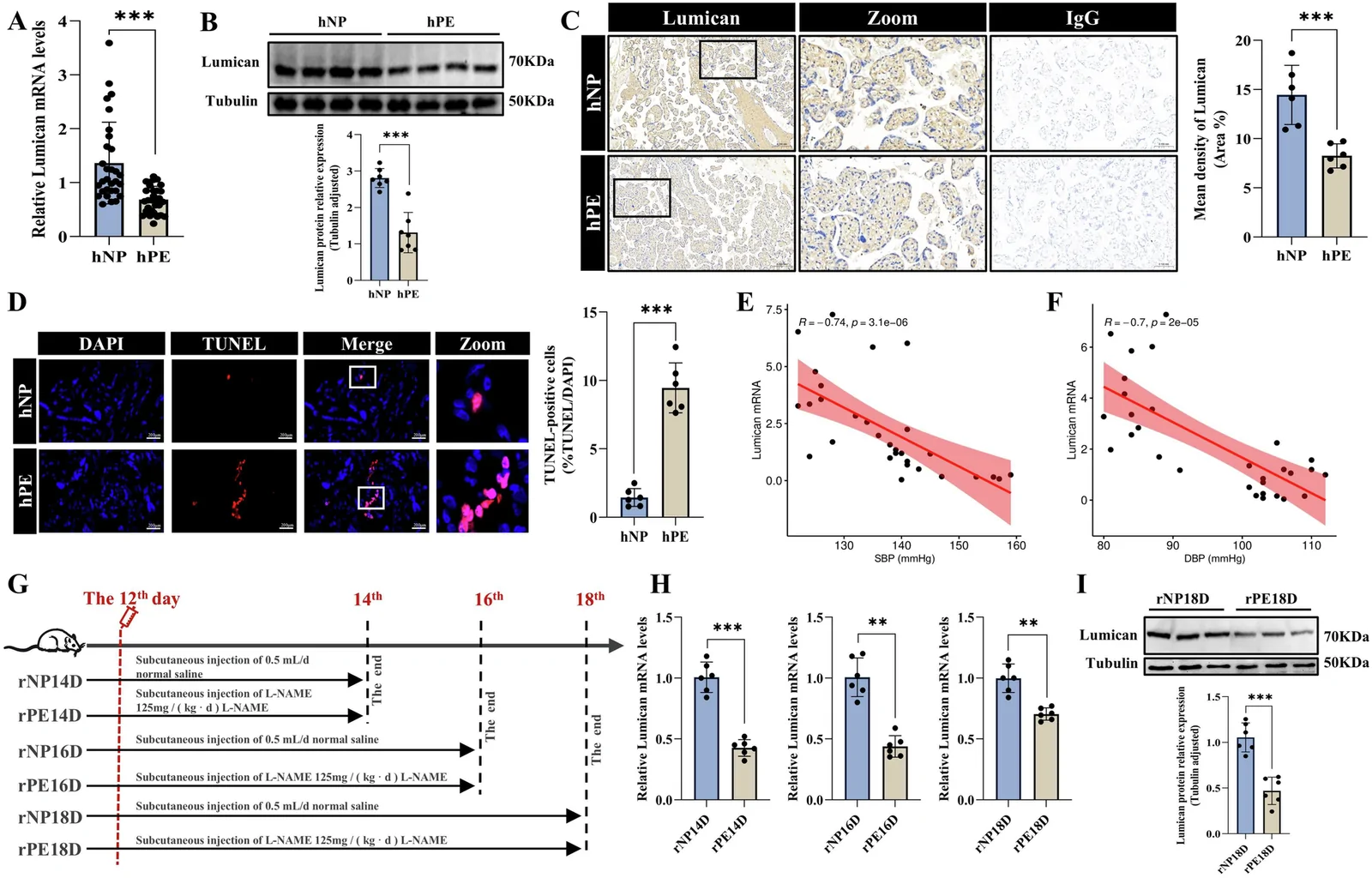
Expression of lumican in PE placenta. **A** Expression level of *lumican* mRNA in PE pregnant women placenta (hPE) and term placentas from normal pregnant women (hNP) (*n* = 30); **B** Expression level of lumican protein in hPE and hNP (*n* = 7); **C** IHC staining; **D** TUNEL-positive cells in placental tissue. The specificity of TUNEL staining was validated using appropriate negative controls (omission of TdT enzyme), shown in Fig. S2A; **E** Spearman correlation analysis between *lumican* mRNA and systolic blood pressure (SBP); **F** Spearman correlation analysis between *lumican* mRNA and diastolic blood pressure (DBP). **G** L-NAME-induced PE rat model. rNP14D: Rat at Gestational Day 14; rPEP14D: PE Rat at Gestational Day 14; rNP16D: Rat at Gestational Day 16; rPE16D: PE Rat at Gestational Day 16; rNP18D: Rat at Gestational Day 18; rPE18D: PE Rat at Gestational Day 18. **H** Expression level of *lumican* mRNA in rat placenta. *n* = 6; **I** Expression level of lumican protein in rat placenta. *n* = 6. Data are presented as the mean ± SEM. Statistical significance was determined by unpaired t-test. *P < 0.05, **P < 0.01, ***P < 0.001, ****P < 0.0001.

RT-qPCR, Western blot, and immunohistochemical analyses confirmed that both protein and mRNA expression levels of lumican were considerably downregulated in the placentas of PE patients (hPE) in comparison with those of normal pregnant women (hNP) (Fig. 3A–C). The TUNEL assay results demonstrated a significant increase in apoptosis in the placental tissues of individuals with PE, as evidenced by a markedly higher ratio of TUNEL-positive cells (Figs. 3D and S2A). Spearman analysis revealed a significant correlation between *lumican* mRNA and both systolic blood pressure and diastolic blood pressure (Fig. 3E, F).

Due to the clinical characteristics of PE, hPE placentas were typically collected around 34 weeks of gestation, whereas hNP samples were mostly obtained at 39 weeks (Table S1). Given our observation that lumican expression increases with gestational age, this gestational age mismatch may confound the comparison of lumican expression levels. To reduce this confounding effect and strengthen our conclusions, we established an animal model for further validation. A rat PE (rPE) model was successfully developed through L-NAME administration (Fig. 3G). The findings revealed a significant elevation in urinary protein content at 14, 16, and 18 days of gestation (Fig. S2B), along with an increase in systolic blood pressure at 18 days (Fig. S2C) in the rPE group, thus confirming the successful development of the PE model. At 18 days of gestation, the placental weight in the rPE group was significantly lower than that in the rNP group (Fig. S2D). In the rPE group, the limbs of the fetal rats turned black, and the placenta appeared darker in color (Figure S2E). HE staining results demonstrated that with the progression of gestation, trophoblast cells in the rNP group gradually showed a clearer and more regular morphology. In comparison, trophoblast cells in the rPE group appeared increasingly irregular. At comparable gestational stages, placentas in the rPE group exhibited severe ischemic injury characterized by trophoblast vacuolar necrosis and a significant decrease in villous capillaries compared to the rNP group (Fig. S2F). RT-qPCR and Western blot showed that the expression of lumican mRNA and protein in the placenta of the rPE group was lower in comparison to the rNP group at the same gestational age (Fig. 3H, I). The abovementioned results collectively indicate that lumican expression was considerably downregulated in PE placentas, both in human and rat placental tissues.

### Significant changes in the PI3K/AKT and P53 signaling pathway in PE placenta

**Fig. 4:**
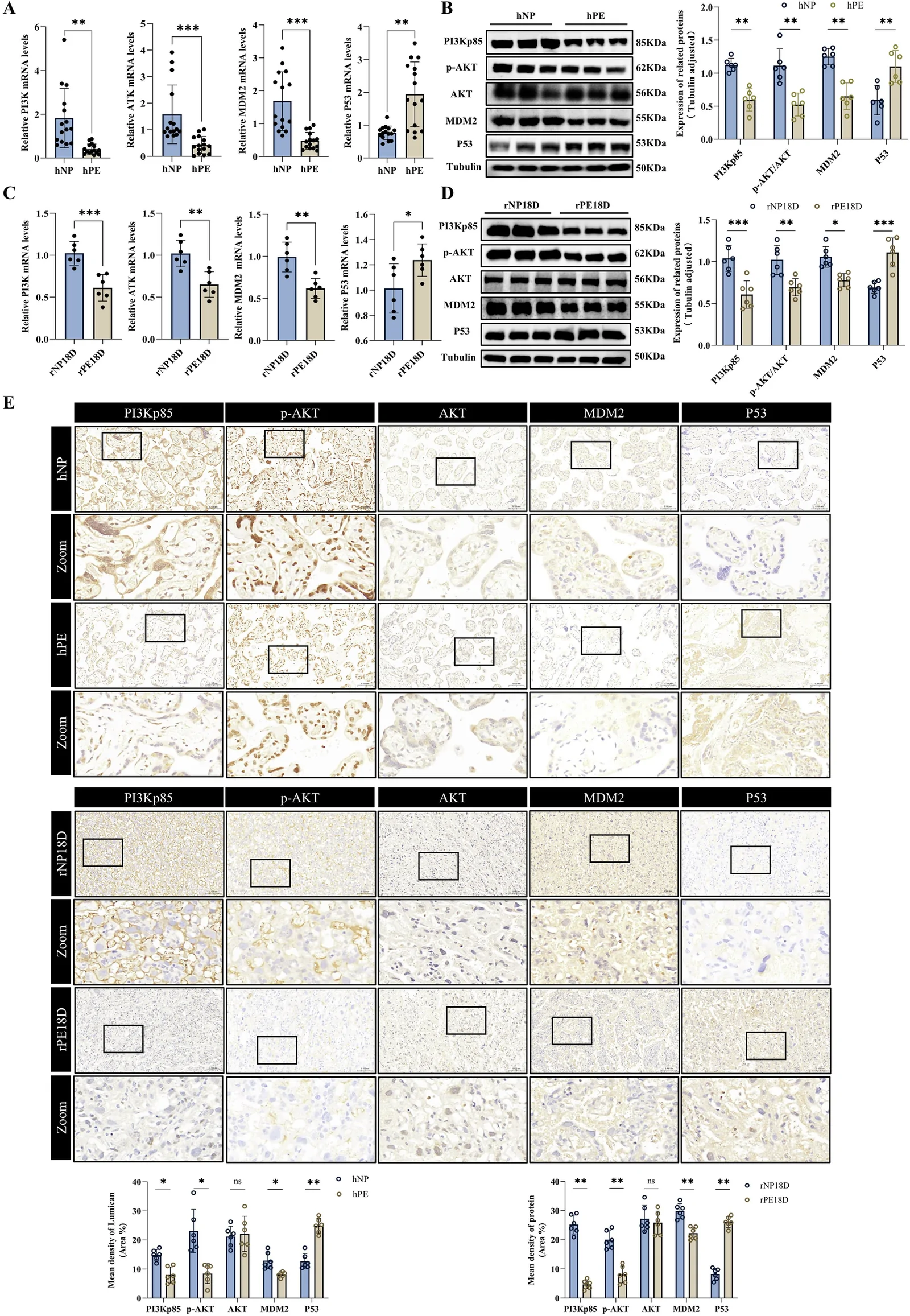
Significant changes in the PI3K/AKT and P53 signaling pathway in PE placenta. **A** Differential mRNA expression levels of *PI3K, AKT, MDM2*, and *P53* was observed in the placentas of PE patients (hPE) in comparison with those of normal pregnant women (hNP). *n* = 15; **B** Differential protein expression levels of PI3K, AKT, MDM2, and P53 was observed in the placentas of hPE in comparison with those of hNP; **C** Differential mRNA expression levels of *PI3K, AKT, MDM2*, and *P53* was observed in the placentas of rPE in comparison with those of rNP; **D** Differential protein expression levels of PI3K, AKT, MDM2, and P53 was observed in the placentas of rPE in comparison with those of rNP; **E** Immunohistochemical analysis of PI3K, AKT, MDM2, and P53 in human and rat placentas. Specificity was confirmed using a matched isotype control antibody, as shown in Supplementary Fig. S7A-C. The scale bar is 200 μm. n = 6. Data are presented as the mean ± SEM. Statistical significance was determined by unpaired t-test. *P < 0.05, **P < 0.01, ***P < 0.001, ****P < 0.0001.

Transcriptomic results indicated that there were 644 up-regulated genes and 968 down-regulated genes in the rPE group in comparison to the rNP group (Figs. S3A and S3B), among which the *lumican* gene was considerably down-regulated in the rPE group (Fig. S3C). GO and KEGG enrichment analyses demonstrated the genes to be highly enriched in the PI3K/AKT signaling pathway (Fig. S3D and S3E), and GSEA analysis shows that the PI3K/AKT signaling pathway is significantly downregulated in the rPE group (Fig. S3F). Analysis of human placental samples from PE patients revealed a significant reduction in PI3K, AKT, and MDM2 expression and an increase in P53 expression, as demonstrated by RT-qPCR and Western blot analyses (Fig. 4A, B). Similarly, altered PI3K, AKT, P53, and MDM2 expression patterns were observed in placentas from PE model rats (rPE) compared to the rNP group (Fig. 4C and D). Immunohistochemical staining further confirmed these findings, showing decreased PI3K and MDM2 protein expression, elevated P53 protein levels, and reduced AKT phosphorylation in the PE group compared to controls (Figs. 4E and S4). The results demonstrate that the PI3K/AKT signaling pathway is down-regulated in PE placenta, and the P53 signaling pathway is up-regulated in PE placenta.

### Lumican downregulation inhibits trophoblast proliferation, migration, and invasion while promoting cell apoptosis

**Fig. 5:**
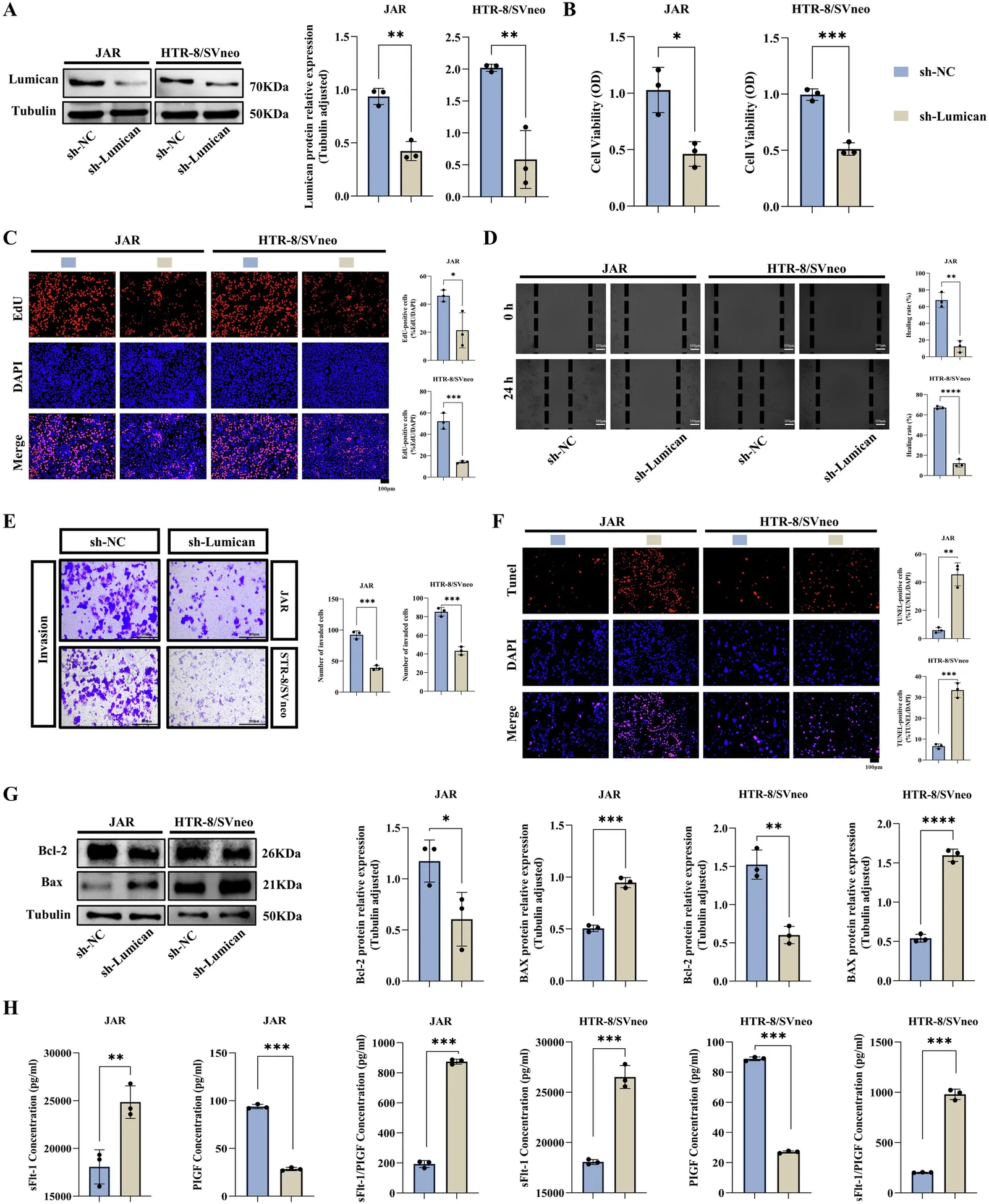
Lumican downregulation inhibits trophoblast proliferation, migration, and invasion while promoting cell apoptosis. **A** Expression levels of Lumican protein; **B** Cell viability of HTR-8/SVneo cells and JAR cells; **C** EdU-positive cells in HTR-8/SVneo cells and JAR cells; **D** Migration of HTR-8/SVneo cells and JAR cells; **E** Invasion capability of HTR-8/SVneo cells and JAR cells; **F** TUNEL-positive cells in HTR-8/SVneo cells and JAR cells; **G** Expression levels of Bcl-2 and Bax proteins. **H** Expression level of sFlt-1, PlGF and sFlt-1/PlGF in HTR-8/SVneo cells and JAR cells, *n* = 3. Data are presented as the mean ± SEM. Statistical significance was determined by unpaired t-test. *P < 0.05, **P < 0.01, ***P < 0.001, ****P < 0.0001.

JAR and HTR-8/SVneo trophoblast cell lines were loaded with sh-Lumican, with transfection efficiency confirmed by Western blot (Fig. 5A). Cell viability was significantly reduced following sh-Lumican transfection (Fig. 5B). EdU incorporation assays revealed decreased proliferation in both JAR and HTR-8/SVneo cells in the sh-Lumican group in comparison to the sh-NC control group (Fig. 5C). Wound healing assays demonstrated reduced migratory capacity in lumican-deficient cells (Fig. 5D), while Transwell assays confirmed impaired invasion ability (Fig. 5E). TUNEL assays indicated an evident increase in apoptosis in the sh-Lumican group compared to controls (Figs. 5F and S5A). Western blot analysis further supported these findings, with significantly decreased Bcl-2 and increased Bax expression in the *lumican*-silenced cells (Fig. 5G). These results suggest that lumican downregulation compromises trophoblast cell viability, proliferation, migration, and invasion, while promoting apoptotic activity. Consistent with established associations between elevated sFlt-1/PlGF ratios and PE, cells with *lumican* knockdown displayed significantly increased sFlt-1 levels and sFlt-1/PlGF ratios, along with reduced PlGF expression (Fig. 5H).

### Effects of lumican downregulation on trophoblast function and the PI3K/AKT and P53 signalling pathway after co-treatment with 740 Y-P

Given that sh-Lumican-transfected JAR and HTR-8/SVneo cells exhibited significantly increased P53 protein expression and a marked reduction in PI3K, p-AKT/AKT, and MDM2 protein levels in comparison to the sh-NC group (Fig. S5B), the effects of lumican downregulation on trophoblast function and associated signaling pathways were further evaluated following co-treatment with the PI3K agonist 740 Y-P. These findings suggest that lumican modulates trophoblast cell behavior via the PI3K/AKT and P53 signaling pathways, contributing to the pathophysiology of PE.

**Fig. 6:**
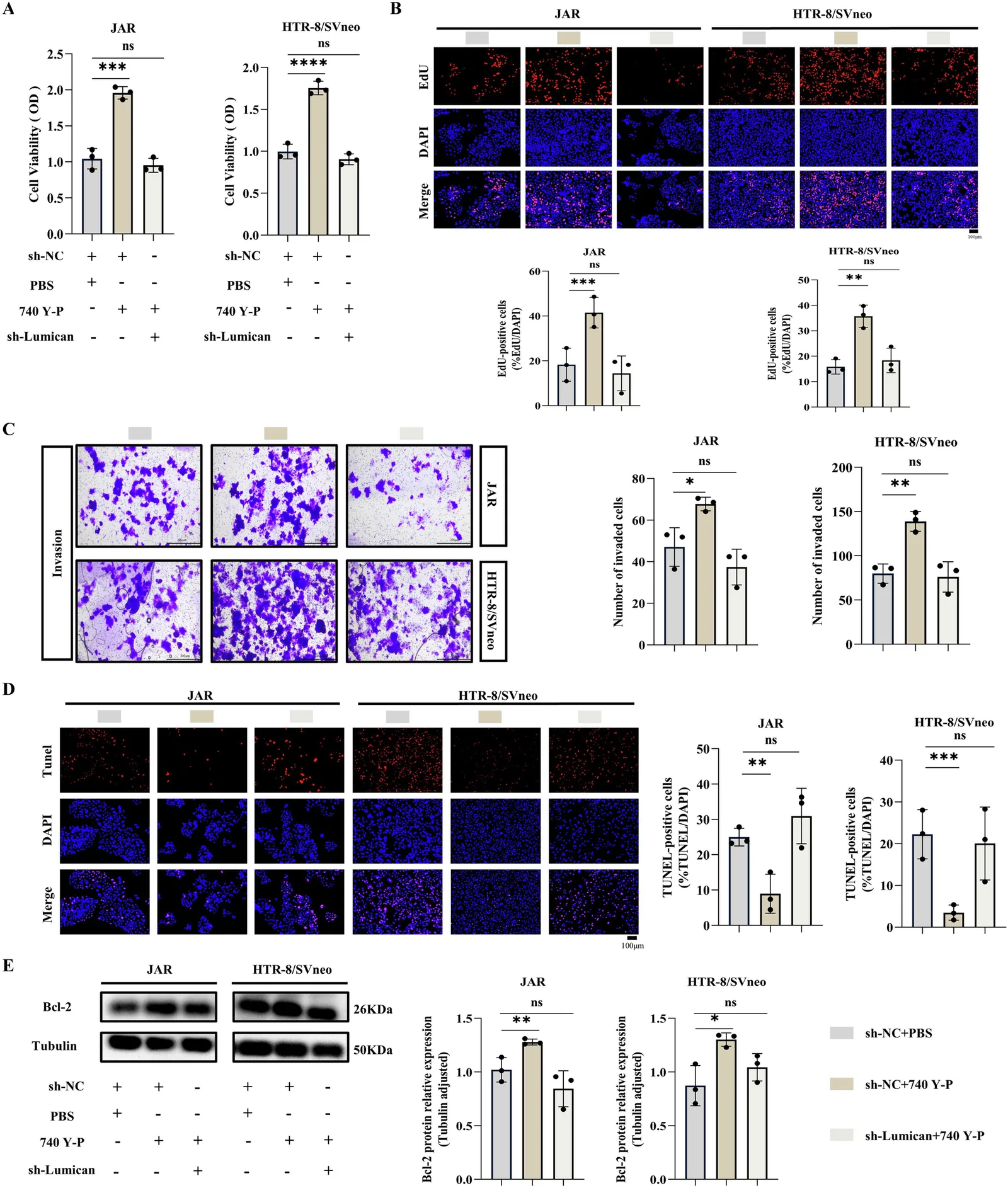
Effect of lumican downregulation combined with 740 Y-P on trophoblast function. **A** Cell viability of HTR-8/SVneo cells and JAR cells; **B** EdU-positive cells in HTR-8/SVneo cells and JAR cells; **C** Invasion ability of HTR-8/SVneo cells and JAR cells; **D** TUNEL-positive cells in HTR-8/SVneo cells and JAR cells; **E** Expression levels of Bcl-2 protein. *n* = 3. Data are presented as the mean ± SEM. Statistical significance was determined by one-way ANOVA with Tukey’s post hoc test for multiple comparisons. *P < 0.05, **P < 0.01, ***P < 0.001, ****P < 0.0001.

As illustrated in Fig. 6A, co-treatment experiments were conducted in JAR and HTR-8/SVneo cells. The CCK-8 assay demonstrated that cell viability was considerably increased in the sh-NC + 740 Y-P group than in the sh-NC + PBS group. However, the viability in the sh-Lumican + 740 Y-P group was reduced and did not differ significantly from that in the sh-NC + PBS group. Similarly, the EdU assay demonstrated significantly enhanced proliferation in the sh-NC + 740 Y-P group relative to the sh-NC + PBS group, while the proliferation rate in the sh-Lumican + 740 Y-P group was reduced and revealed no significant difference from the control (Fig. 6B).

Transwell invasion assays revealed significantly increased invasion in the sh-NC + 740 Y-P group compared to the sh-NC + PBS group. On the other hand, no significant difference was observed in the invasion capacity of the sh-Lumican + 740 Y-P group relative to the sh-NC + PBS group (Fig. 6C). TUNEL assays showed a significant reduction in the number of apoptotic cells in the sh-NC + 740 Y-P group; however, apoptosis levels in the sh-Lumican + 740 Y-P group remained comparable to the sh-NC + PBS group (Figs. 6D and S5C).

Western blot analysis confirmed these observations, revealing increased expression of the anti-apoptotic protein Bcl-2 in the sh-NC + 740 Y-P group. No significant change in Bcl-2 expression was detected in the sh-Lumican + 740 Y-P group (Fig. 6E).

RT-qPCR and Western blot showed that, in the sh-NC + 740 Y-P group, P53 expression was significantly decreased, while the expressions of PI3K, AKT, and MDM2 were elevated compared to the sh-NC + PBS group. On the other hand, no considerable variations in PI3K, AKT, P53, or MDM2 expression were observed in the sh-Lumican + 740 Y-P group than in the sh-NC + PBS group (Fig. S6A and S6B).

The findings reveal that the PI3K agonist 740 Y-P effectively activates the PI3K/AKT and P53 signaling pathways in JAR and HTR-8/SVneo cells. However, this impact is counteracted by lumican downregulation, suggesting that lumican is essential for maintaining trophoblast responsiveness to PI3K/AKT and P53 signaling activation.

### Lumican deficiency induces PE-like symptoms in pregnant rats

**Fig. 7:**
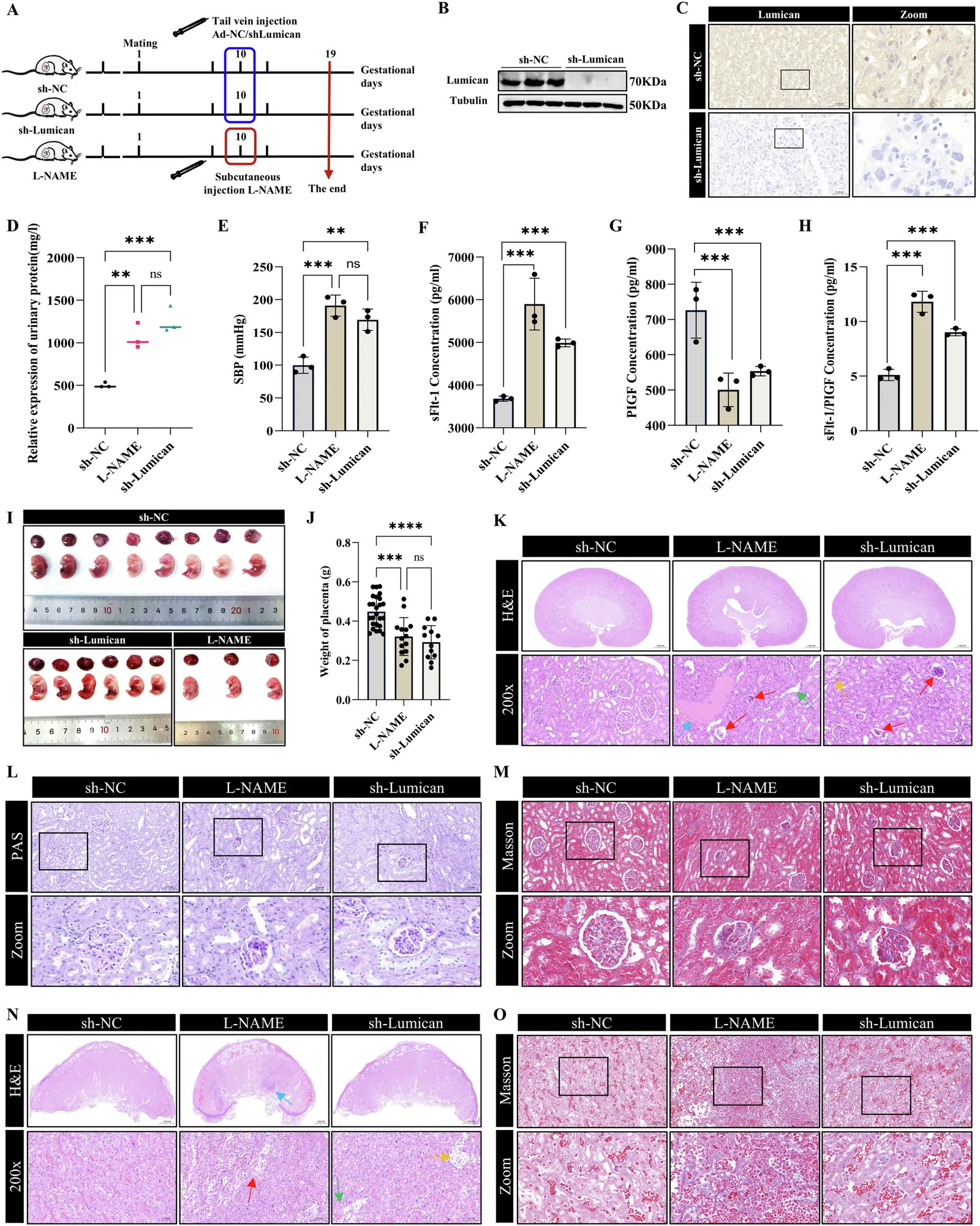
Lumican deficiency induces PE-like symptoms in pregnant rats. **A** Development of the sh-Lumican rat model; **B** Protein expression levels of lumican in the rat placenta; **C** In situ expression levels of lumican in the rat placenta. Specificity was confirmed using a matched isotype control antibody, as shown in Fig. S7D; **D** Proteinuria levels; **E** Systolic blood pressure (SBP); **F** Expression levels of sFlt-1 in rat serum; **G** Expression levels of PlGF in rat serum; **H** Expression levels of sFlt-1/PlGF in rat serum; **I** Placenta and fetal rats; J: Placental weight; **K** HE staining in rat kidney tissue; **L** PAS staining in rat kidney tissue; **M** Masson staining in rat kidney tissue; **N** HE staining in rat placental tissue; **O** Masson staining in rat placental tissue. The scale bar is 200 μm; *n* = 3. Data are presented as the mean ± SEM. Statistical significance was determined by one‑way ANOVA with Tukey’s post hoc test. ***P < 0.001, **P < 0.01, *P < 0.05, n.s. not significant.

To further investigate whether lumican deficiency induces PE-like symptoms, a pregnant rat model was developed by administering adenovirus-mediated sh-Lumican *via* tail vein injection on gestational day 10 (GD10) (Fig. 7A). Lumican expression in the placenta, assessed on GD19, was significantly reduced after adenoviral infection (Fig. 7B, C). Lumican deficiency markedly increased 24 h proteinuria levels (Fig. 7D) and elevated systolic blood pressure (Fig. 7E) in pregnant rats. As illustrated in Fig. 7F–H, the L-NAME positive control group and the sh-Lumican group of pregnant rats revealed enhanced plasma sFlt-1 levels and decreased plasma PlGF levels. Moreover, the sh‑Lumican group exhibited a significant decrease in the number of fetuses compared to the sh‑NC group (Fig.7I), suggesting fetal resorption likely resulting from severe placental insufficiency. Both the sh-Lumican and L-NAME groups showed a considerable decrease in placental weight compared to the sh-NC group (Fig. 7J). Renal histopathological examination in the sh-Lumican group demonstrated glomerular contraction, interstitial edema, and evident renal ischemia. (Fig. 7K-M). Moreover, histological analysis of placental tissues revealed reduced labyrinthine vascular branching, widened intervillous spaces, and fibrous matrix deposition in the sh-Lumican group compared to the sh-NC group (Fig. 7N, O). The results demonstrate that lumican deficiency in pregnant rats induces PE-like symptoms, adversely affecting both placental and fetal development.

### Lumican deficiency alters placental ultrastructure and the PI3K/AKT and P53 signaling pathway

**Fig. 8:**
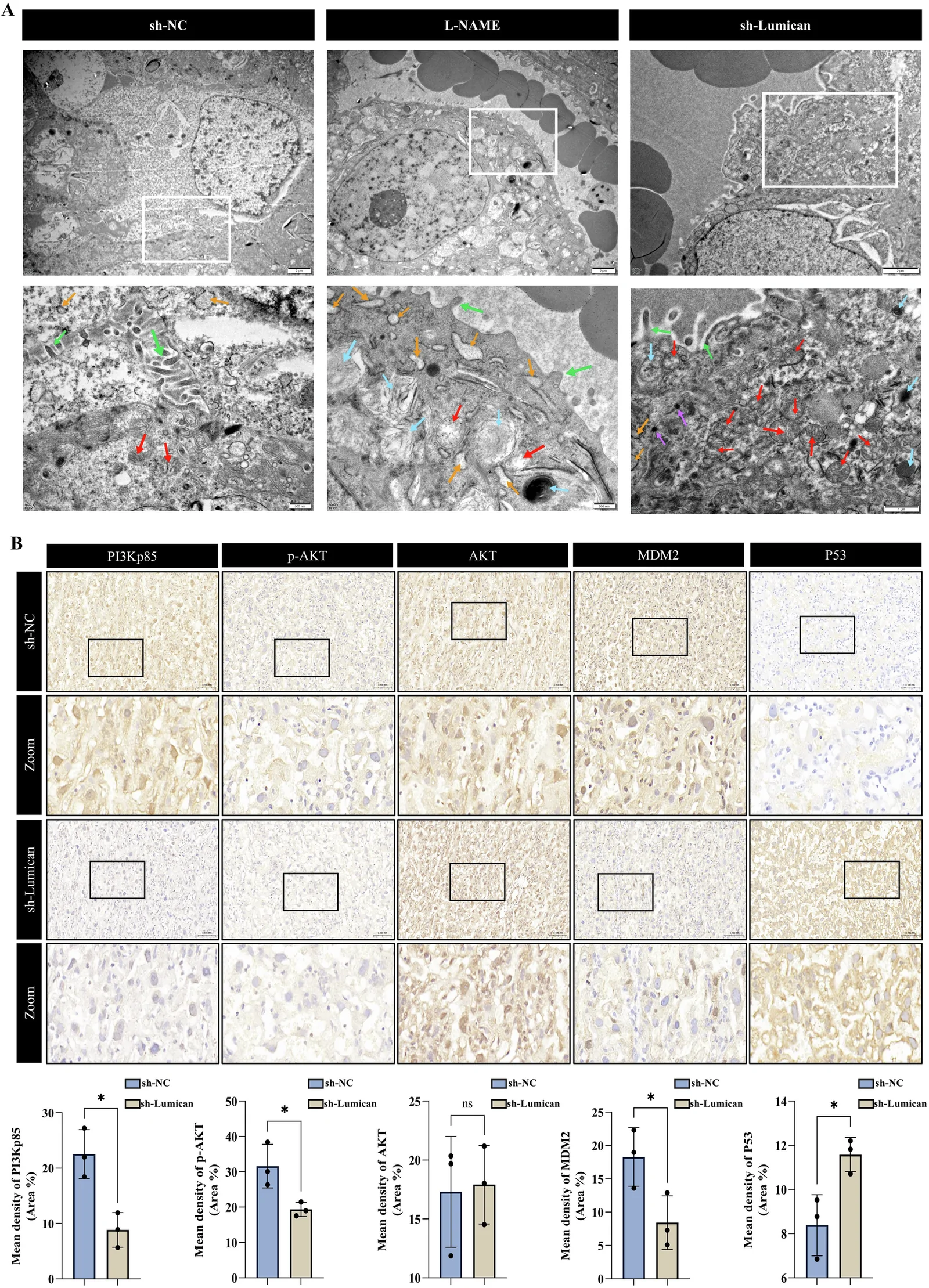
Effect of lumican knockdown on placental ultrastructure and related protein expression. **A** TEM of trophoblasts in the rat placenta: red arrow—mitochondria; orange arrow—rough endoplasmic reticulum; green arrow—microvilli on the cell membrane surface; blue arrow—autophagic lysosomes and primary lysosomes; purple arrow—trophoblastic cell junction. **B** In situ expression levels of PI3K, AKT, p-AKT, MDM2, and P53 in rat placenta. Specificity was confirmed using a matched isotype control antibody, as shown in Fig. S7E. The scale bar is 200 μm; *n* = 3. Data are presented as the mean ± SEM. Statistical significance was determined by unpaired t-test. ***P < 0.001, **P < 0.01, *P < 0.05, n.s. not significant.

The results of Transmission electron microscopy (TEM) of placental tissue in the sh-NC group revealed that the mitochondrial matrix density in trophoblast cells was moderate, with no evident edema observed. The cristae were partially visible and arranged in a plate-like manner. Ribosome attachment was observed on the cytoplasmic surface of the endoplasmic reticulum, and the microvilli on the cell membrane were numerous and closely arranged, showing no significant damage. On the other hand, the results from the L-NAME group indicated a significant decrease in mitochondrial matrix density, with the matrix exhibiting marked edema. The cristae showed severe fracture and dissolution, with no normal cristae structure present. The spaces between endoplasmic reticulum vesicles were widened, and the endoplasmic reticulum was moderately dilated, accompanied by ribosomal degranulation on the cytoplasmic surface. The microvilli on the cell membrane were fewer in number and sparsely arranged, with some microvilli disappearing. Furthermore, vacuolar structures were present, containing irregular floccules and some in a medullary shape. There is a slight decrease in mitochondrial matrix density in the sh-Lumican group, with mild edema observed in the matrix and partial dissolution of the cristae. The spacing between the rough endoplasmic reticulum cisternae is widened, and the endoplasmic reticulum appears mildly dilated, with ribosomes attached to the cytoplasmic surface. Microvilli are present on the cell membrane surface, although their quantity is relatively low and arranged sparsely. Autophagic lysosomes and primary lysosomes are evident. Moreover, bridge connections between trophoblast cells are visible. These findings suggest that sh-Lumiacn can induce trophoblast and mitochondrial damage in the placentas of pregnant rats (Fig. 8A). In the placenta, compared to the sh-Lumican and sh-NC groups, immunohistochemical results showed a considerable reduction in the PI3K and MDM2 proteins expression, a reduction in AKT phosphorylation, but a considerable enhancement in the P53 protein expression (Fig. 8B).

## Discussion

Every year, PE causes over 500,000 fetal and 70,000 maternal deaths worldwide, making it a considerable contributor to perinatal and maternal mortality and morbidity^26–28^. Currently, placental STB stress is recognized as a pivotal factor in the PE pathogenesis^29,30^. Comprehensive elucidation of the specific determinants and molecular mechanisms underlying STB stress holds significant importance for effective preventive and therapeutic strategies for PE.

STB dysfunction may be associated with changes in the composition or function of the ECM. Lumican, a prominent member of ECM family, is known to modulate diverse cellular functions^31^. Evidence suggests that lumican promotes in vivo tumorigenesis and in vitro cancer cell migration, invasion, and proliferation^32^. Lumican enhances the progression of hepatocellular carcinoma (HCC) by suppressing the P53 signaling pathway^33^. Earlier findings from our laboratory showed that depleting lumican substantially reduces migration and proliferation in HTR‑8/SVneo trophoblasts; however, the precise signaling mechanism driving this phenotype was not resolved.

Our study provides the first systematic evidence for the dynamic and essential role of lumican in placental development and PE pathogenesis. We observe that lumican expression increases progressively during normal pregnancy, coinciding with physiological placental growth and enhanced anti-apoptotic signaling, including PI3K/AKT activation and P53 suppression. In contrast, lumican is markedly downregulated in PE placentas across gestation stages, correlating with core clinical features such as hypertension. These findings establish lumican dysregulation as a key molecular determinant of trophoblast dysfunction in PE.

Recently, considerable research has been directed toward elucidating the contribution of the PI3K/AKT pathway in the pathophysiology of PE. Studies have reported that Syntaxin 2 expression is downregulated in the placental tissues of PE patients. Syntaxin 2 activates the AKT signaling pathway by specifically binding to the p85 subunit of PI3K and recruiting it to the cell membrane^22^. Moreover, it promotes trophoblast invasion *via* the PI3K/AKT/eNOS signaling cascade. Progesterone has been shown to inhibit PE by enhancing trophoblast invasion by stimulating the PI3K/AKT pathway^34^. Studies indicate that Olfactomedin-like 3 (OLFML3) may attenuate PE progression by inhibiting the PI3K/AKT pathway, leading to apoptosis in extravillous trophoblast cells^35^. Furthermore, decreased expression of Transforming Acidic Coiled-Coil Protein 3 (TACC3) has been observed in PE placentas. Suppression of *TACC3* expression impairs the PI3K/AKT signaling pathway, thus impeding the movement and invasion of HTR8/SVneo cells^36^. The MDM2/P53 signaling pathway has also been involved in the pathogenesis of PE. Reduced protein disulfide isomerase A3 (PDIA3) expression in placental trophoblasts regulates the MDM2/P53 pathway, leading to increased trophoblast apoptosis^24^. Research has shown that elevated P53 levels in human umbilical vein endothelial cells from PE patients activate the *Bax* gene while suppressing *Bcl-2* expression. This results in an increased Bax/Bcl-2 ratio, thus promoting apoptosis. Trophoblasts in PE undergo apoptosis due to the upregulated P53 and Bax protein expression and a downregulation of MDM2 protein expression^37,38^.

In contrast to previous studies which predominantly examined individual signaling pathways, our work demonstrates that lumican sustains trophoblast homeostasis by concurrently activating the PI3K/AKT pathway and inhibiting P53 signaling, thereby establishing a central regulatory node essential for cellular survival and function. Comprehensive RNA sequencing analysis of rat placental tissues revealed substantial alterations in the PI3K/AKT and P53 signaling pathways during placental development. The PE rat model and placental specimens obtained from PE patients also showed significant downregulation of AKT, PI3K, and MDM2 expression levels and marked upregulation of P53 expression. These outcomes indicate that the PI3K/AKT and P53 signaling cascade is a crucial regulatory mechanism in the onset and progression of PE throughout placental maturation. Critically, we found that the disruption of these pathways originates from diminished lumican expression. Reduced lumican attenuates PI3K/AKT signaling and lowers MDM2 levels, which in turn releases the brake on P53 and initiates its apoptotic program, while also compromising trophoblast proliferation and invasion. We confirmed this lumican-driven signaling cascade in cellular models (via rescue with the PI3K agonist 740Y‑P), PE animal models, and clinical placental samples.

Taken together, our work identifies lumican as a critical upstream regulator in preeclampsia pathogenesis. Mechanistically, lumican deficiency triggers a signaling cascade wherein suppressed PI3K/AKT activity and diminished MDM2 converge to stabilize p53. This in turn activates mitochondrial apoptosis, compromising trophoblast function, impairing placental invasion, ultimately contributing to the maternal clinical manifestations of PE. Thus, lumican downregulation contributes to disease progression through this defined molecular pathway.

Our mechanistic insights suggest novel therapeutic avenues for PE that go beyond current symptom-focused management. We establish lumican as a pivotal regulator of trophoblast homeostasis and placental integrity. Lumican deficiency disrupts trophoblast function, leading to impaired proliferation, suppressed migration, and reduced cell survival. Therefore, targeted strategies to restore lumican signaling—via protein supplementation, peptide mimetics, or transcriptional modulation—could rebalance the PI3K/AKT and P53 pathways and support placental repair. This approach, centered on reviving the placenta’s intrinsic developmental potential, aligns closely with the principles of regenerative medicine. Clinically, lumican expression in placental tissue shows significant correlation with both systolic and diastolic blood pressure measurements. Experimental lumican knockdown consistently elevates the sFlt-1/PlGF ratio, reflecting the biomarker profile used in clinical risk assessment. These parallel findings identify lumican as a promising diagnostic marker for advancing preeclampsia management.

Our study has certain limitations that warrant consideration. First, because PE often leads to preterm delivery, our clinical placental samples could not be precisely matched for gestational age. This introduces the possibility that some of the variation in lumican expression may be attributable to developmental stage rather than pathology alone. While our animal studies reveal a significant difference in lumican expression between gestational age-matched PE rats and normal control rats, confirming these findings in carefully gestational age-matched human placenta cohorts remains an essential next step. Second, the experimental work relied largely on two established trophoblast cell lines (JAR and HTR‑8/SVneo). While these models are widely used, they differ genetically and functionally from primary trophoblasts or human trophoblast stem cells, which more closely resemble the in vivo state. Therefore, future studies utilizing primary human trophoblasts or human trophoblast stem cells will be essential to validate our findings and further elucidate the precise role of lumican in trophoblast function in *vivo*. Finally, although we have linked lumican to PI3K/AKT and P53 signaling, the exact mechanism by which this extracellular matrix protein engages cell surface receptors to initiate downstream events requires further investigation.

**Fig. 9:**
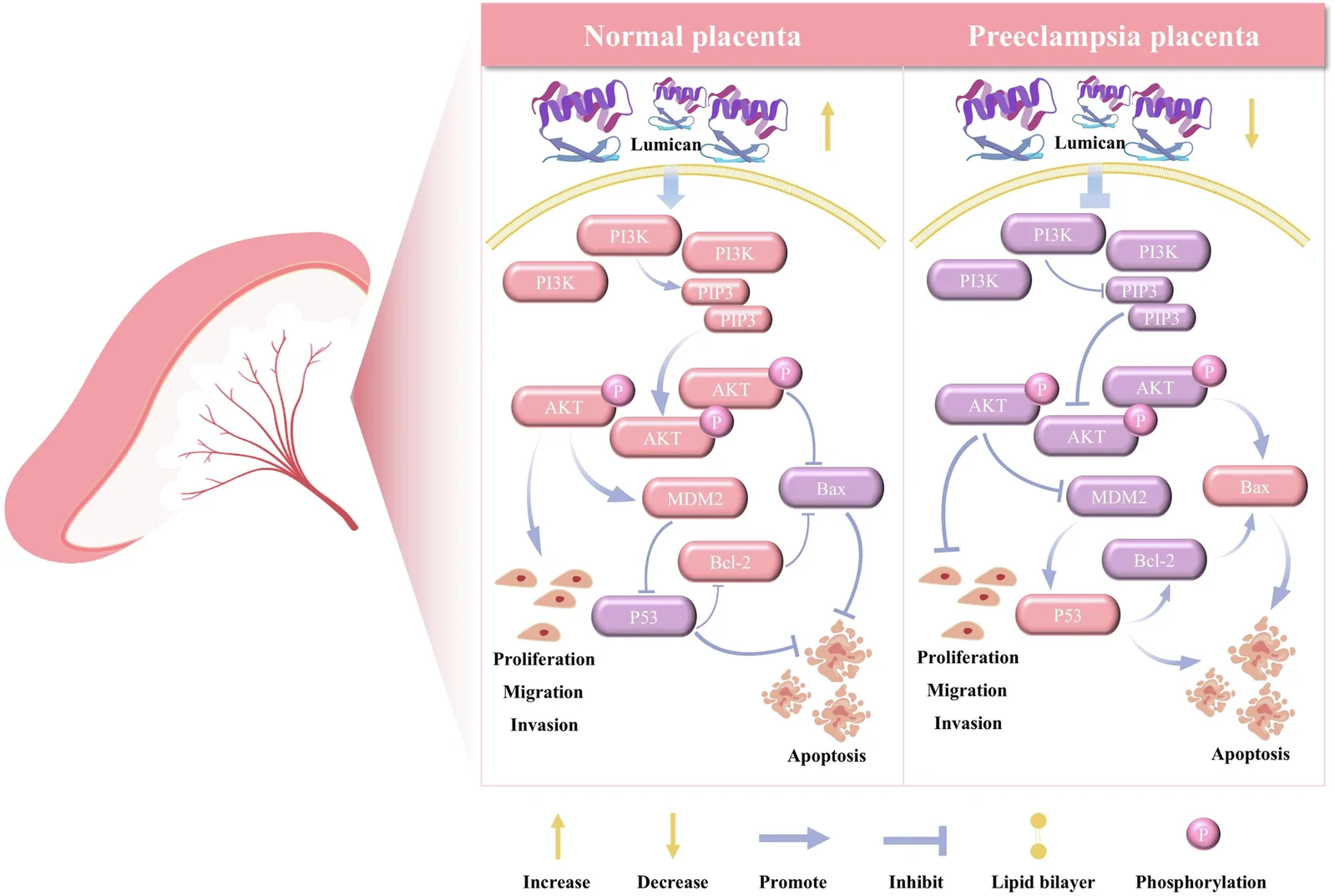
Lumican Regulates PI3K / AKT and P53 Signaling Pathways in PE. Schematic illustration of the differential signaling pathways in normal placenta and PE placenta. In normal placenta (left panel), lumican activates the PI3K/AKT pathway, which subsequently upregulates MDM2. This promotes trophoblast proliferation, migration, and invasion, while simultaneously suppressing apoptosis. In PE placenta (right panel), lumican deficiency leads to inhibition of PI3K/AKT signaling. The reduced AKT activity relieves the suppression on P53, resulting in increased Bax expression. This signaling shift promotes trophoblast apoptosis and inhibits proliferation, migration, and invasion, ultimately contributing to the pathogenesis of PE. In this schematic, pink‑colored proteins indicate upregulation or increased activity, while purple‑colored proteins indicate downregulation or decreased activity.

This work defines lumican as a critical regulator of placental integrity and PE progression. During healthy pregnancy, rising lumican levels sustain trophoblast function through PI3K/AKT activation and concomitant P53 inhibition. In PE, however, lumican deficiency destabilizes this signaling equilibrium, triggering trophoblast failure and placental impairment (Fig. 9). These insights advance the pathophysiological framework of PE and position lumican as a translational target for diagnostic and regenerative approaches.

## Methods

### Clinical sample collection

Placental tissues were collected immediately after delivery from patients diagnosed with preeclampsia (hPE, *n* = 30) and matched normotensive controls (hNP, *n* = 30). The PE cohort predominantly comprised preterm PE cases (mean gestational age at delivery: ~34 weeks; see Table S1 for full demographics). The inclusion criteria for the PE group were considered as systolic blood pressure ≥ 140 mmHg and/or diastolic blood pressure ≥ 90 mmHg, in combination with proteinuria of ≥ 1+ on dipstick testing or 24 h urinary protein excretion ≥ 300 mg. After delivery, placental tissues were immediately processed by washing with sterile saline. Central placental sections were excised and stored at −80 °C. In addition, early-gestation chorionic tissue samples (designated as first-trimester placenta hFTP, *n* = 30) were collected from women with intrauterine singleton pregnancies (gestational age: 53–77 days) undergoing elective termination for non-medical reasons. All hFTPs samples were confirmed by ultrasonography and subsequent histological evaluation to be morphologically normal early placental (chorionic) tissues, with no evidence of pathology. These samples serve as the early developmental control for comparison with term placentas. Informed consent was acquired from all subjects before sample collection. All procedures were conducted under ethical standards, and the project received approval from the Institutional Review Board of Jining Medical University (Ethical Approval Number: 2021-YX-ZR-028), with applicable regulations and ethical guidelines.

### Establishment of the PE rat model

A total of 18 male (350–450 g) and 18 female (240–260 g) Sprague-Dawley (SD) rats were obtained under the animal production license number SCXK (Lu) 20190003. All animals were obtained from Jinan Pengyue Laboratory Animal Breeding Co., Ltd. All animal care and experimental procedures were conducted in accordance with ethical guidelines and were approved by the Ethics Committee of Jining Medical University (Approval No. 2021-DW-ZR-052). The SD rats were maintained in a regulated setting (12 h light/dark cycle; 25 °C; 45–55% humidity) and had free access to sterilized drinking water and food. The estrous cycle phases of the female SD rats were monitored through vaginal cytology. After the identification of the appropriate reproductive stage, mating was performed. Confirmation of pregnancy was based on the vaginal smears or the presence of vaginal plug shedding, designating day 1 of gestation. A rat PE model was developed employing Nω-Nitro-L-arginine methyl ester (L-NAME)^39–41^. According to the protocol of Zeng et al.^42–44^, pregnant rats were randomized into the rPE and rNP groups. The rPE group received subcutaneous injections of L‑NAME (125 mg/kg/day, dissolved in 0.5 mL of solution) starting on the 12th day of gestation. The next rNP group was administered the same saline volumes (0.5 mL/day) via subcutaneous injection. Urinary proteins were quantified using a quantitative assay kit based on the Coomassie Brilliant Blue (CBB) method at 595 nm absorbance. Moreover, heart rate, systolic blood pressure, and diastolic blood pressure were evaluated through a non-invasive tail-cuff system to measure hemodynamic parameters through the caudal arteries. Tissue specimens were subsequently collected on GD14, GD16, and GD18. At each time point, rats were anesthetized with isoflurane (induction: 4–5% in oxygen; maintenance: 1.5–2% in oxygen) in an induction chamber, and then euthanized by cervical dislocation to ensure death. From each pregnant rat, two placentas were randomly selected from all available placentas using a random number table: one for molecular biological analyses (qPCR and Western Blot), and another independent placenta for pathological examination.

### The PE rat model was induced by sh-Lumican

Adenoviral vectors encoding *lumican*-targeting shRNA (sh-Lumican) and non-targeting control shRNA (sh-NC) were constructed (Fenghui Biotechnology). Pregnant rats were randomized into L-NAME, sh-Lumican, and sh-NC groups (*n* = 3/ group). Initially, on gestational day 10, the L-NAME group received daily subcutaneous injections of L-NAME (125 mg/kg). Simultaneously, the sh-Lumican and sh-NC groups were administered 2 × 10^9^ PFU of the respective adenoviral vectors *via* tail vein injection. On gestational day 19, non-invasive blood pressure was monitored *via* the tail artery under isoflurane anesthesia. Next, biological samples, including plasma, urine, tissues (placenta and kidney), and fetuses, were harvested. Plasma, urine, and all tissue samples were rapidly frozen in liquid nitrogen and preserved at -80 °C for further analysis, whereas the residual tissues were kept in 4% paraformaldehyde (PFA, BL539A, Biosharp) for histopathological evaluation. Following the manufacturer’s procedures, plasma PlGF and sFlt-1 levels were measured utilizing commercially available ELISA kits.

### Cell culture

JAR and HTR-8 / SVneo cell lines (Fenghui Biotechnology Co., Ltd.) are both recognized in vitro models for PE. Fetal bovine serum (FBS, 10%; Bioind, Israel) was added to the complete growth medium to maintain cell viability, followed by incubating at 37 °C in a 5% CO_2_ environment.

### Cell transfection

The pGPU6/GFP/Neo vector was used to construct the *lumican*-targeting knockdown plasmid. A shRNA sequence specifically targeting human *lumican* (sh-Lumican: 5′-CCAGAATGTAACTGCCCTGAA-3ʹ), selected based on optimal silencing efficiency, was designed. Upon reaching approximately 60% confluence, JAR and HTR-8/SVneo cells were transfected with the pGPU6-human *lumican* shRNA plasmid using LipoFiter™ 3.0 transfection reagent (Hanbio Technology). After transfection, the cells were cultured for 24–72 h before proceeding with experimental procedures.

### ELISA

The concentrations of soluble fms‑like tyrosine kinase‑1 (sFlt‑1) and placental growth factor (PlGF) were measured using species‑specific commercial ELISA kits. For rat serum samples, the Rat sFlt‑1 ELISA Kit (Jianglai Bio. JL48077) and Rat PlGF ELISA Kit (Jianglai Bio. JL11559) were used. For conditioned media from human trophoblast cell lines (JAR and HTR‑8/SVneo), the Human sFlt‑1 ELISA Kit (Jianglai Bio. JL13928) and Human PlGF ELISA Kit (Jianglai Bio. JL23762) were employed. All assays were performed according to the manufacturer’s protocols. Absorbance was measured at 450 nm with a reference wavelength of 570 nm, and concentrations were calculated from standard curves with all samples assayed in duplicate.

### CCK-8 assay

Cells (5 × 10^3^ cells/well) were seeded in 96-well plates and incubated overnight. Following a 24 h plasmid transfection period, 100 μL of a solution containing Dulbecco’s Modified Eagle Medium (DMEM) and Cell Counting Kit-8 (CCK-8, A311, Vazyme) reagent (9:1 ratio) was added per well after aspirating the old medium. Cell viability was assessed by recording the absorbance of the cells at 450 nm after a 2 h incubation period.

### EdU experiment

Cells were grown in 24-well plates (5 × 10^3^ cells/well) and incubated overnight. For cell proliferation labeling, the culture medium was added with 10 μM 5-ethynyl-2′-deoxyuridine (EdU, A412-01, Vazyme) and 10% FBS. After incubation, 100 μL of Click Reaction Mixture was added per well, and the plate was then incubated in the dark for 30 minutes at room temperature (RT). Nuclear staining was performed using DAPI. Fluorescent images were taken using an inverted fluorescence microscope, followed by their analysis while employing ImageJ software.

### Scratch cell assay

Cells (1 × 10^5^ cells/well) were grown in 6-well culture plates, followed by incubation overnight to allow for adhesion. After two washes with PBS, a linear scratch was induced in the monolayer while employing a 10 μL sterile pipette tip to develop a wound healing (scratch assay) model. Reference lines were marked on the bottom of the plate for consistent imaging fields. Bright-field images of the wound area were taken at 0 and 24 h. The relative wound closure area was quantified *via* ImageJ software to evaluate cell migration potential.

### Transwell assay

Transwell chambers were pre-coated with Matrigel matrix. Approximately 100 μL of DMEM was introduced into the upper chamber, while 600 μL of 10% FBS-supplemented DMEM was incorporated into the lower chamber as a chemoattractant. JAR and HTR-8/SVneo cells were seeded (5 × 10^4^ cells/well) into the upper chamber, followed by incubation under standard culture conditions. Thereafter, the chambers were gently rinsed with PBS and preserved with 4% PFA at RT for 30 min. Invaded cells were stained using 1% crystal violet by immersing the transwell chamber in 600 μL of the staining solution for 20 min. Finally, the membranes were mounted with neutral resin, and the stained cells were visualized and quantified.

### TUNEL assay

Cells (5 × 10^3^ cells/well) were cultured onto coverslips placed in 24-well culture plates and incubated overnight. After that, they were fixed with 4% PFA (1 mL/well) for 20 min at RT, and then washed three times with PBS (5 min/ wash). Cells were permeabilized using 0.5% Triton X-100 for 5 min at RT, after which the cells underwent washing with PBS (thrice). After 30 min equilibration in 1X reaction buffer at RT, TUNEL reaction mixture (50 Μl, A113-01, Vazyme) was introduced into each well and incubated at 37 °C in the dark for 60 min. Nuclear staining was performed with DAPI in the dark for 5 min, followed by PBS washing (thrice). Finally, coverslips were mounted using the antifade mounting medium, and fluorescence signals were captured with an inverted fluorescence microscope. Fluorescence signals were quantified using ImageJ software. A negative control for each experiment was performed by omitting the terminal deoxynucleotidyl transferase (TdT) enzyme from the reaction mixture. No specific signal was observed in these negative control sections.

### RT-qPCR

Total RNA was isolated employing the Total RNA Extractor (Trizol, B511311, Sangon). About 1 μg of RNA was reverse-transcribed into cDNA employing the HiScript III RT SuperMix for qPCR (+gDNA wiper, R323, Vazyme) with removal of genomic DNA. qPCR was conducted using a 20 μL reaction volume having 2X AceQ SYBR Green Master Mix, forward and reverse primers (sequences provided in Table S2), and cDNA template. Reactions were conducted with the following thermal profile: 94°C for 5 min; 38 cycles of 94 °C for 45 s, 55 °C for 45 s, and 72°C for 90 s; final extension at 65 °C for 5 s. Reactions were triplicated employing an Applied Biosystems QuantStudio 5 system. The calculation of the Relative gene expression was carried out through the method of the 2^−ΔΔC^t, with β-actin employed as the reference gene.

### Western blot

The preparation of protein extracts from placental tissue and trophoblast cells (human and rat) was carried out using RIPA lysis buffer added with protease inhibitors. Proteins were denatured in Loading buffer at 95 °C for 5 min, and concentrations were measured by BCA assay. Equal contents of protein were separated on 10% SDS-PAGE gels and moved to PVDF membranes, which, following blocking with 5% skim milk in TBST for 2 h at RT, were incubated overnight at 4 °C using primary antibodies (listed in Table S3). Following three TBST washes (10 min each), membranes were probed with HRP-conjugated secondary antibodies (1:1000, Beyotime) for 1 h. Visualization of the bands was carried out while employing an enhanced chemiluminescence detection system (Thermo Fisher), followed by their analysis using ImageJ software. Original, uncropped scans of key blots are provided in Fig. S8.

### Histopathologic study

Kidney and placental tissues were fixed in 4% PFA, followed by graded ethanol and xylene dehydration. The samples were embedded in paraffin and sectioned to prepare paraffin-embedded tissue sections. Histopathological evaluation of human placental tissues and rat placental and renal tissues was performed using HE, Masson’s trichrome, and PAS staining to observe pathological variations. For Immunohistochemistry staining, after antigen retrieval and blocking, tissue sections were incubated overnight at 4°C with specific primary antibodies (listed in Table S3). For each primary antibody, a matched rabbit IgG isotype control (Proteintech Cat # 30000-0-AP) was used at the same protein concentration on parallel sections as a negative control to confirm staining specificity. No specific staining was observed in any isotype control sections. The following day, sections were incubated with appropriate HRP-conjugated secondary antibodies, developed with DAB substrate, and counterstained with hematoxylin.

### RNA sequencing

The RNA concentration and purity were evaluated *via* the NanoDrop 2000 spectrophotometer. Next, mRNA was used as the template for PCR amplification to construct the enriched cDNA library. Sequencing was carried out on the Illumina NovaSeq 6000 platform in paired-end 150 (PE150) mode. A bioinformatics study was conducted *via* the BMKCloud platform (www.biocloud.net), comprising gene function annotation, functional enrichment analysis, structural analysis, differential gene expression (DEG) profiling, and evaluation of library quality. *P*-value ≤ 0.05; Fold Change(FC) ≥ 1.5.

### Transmission electron microscopy (TEM)

The placenta tissue was trimmed into 1 mm³ pieces and fixed in a 2.5% glutaraldehyde solution. It was then dehydrated using a series of anhydrous ethanol concentrations, immersed in propylene oxide overnight, and then embedded and sliced. The sections were stained with 0.5% uranyl acetate and 0.25% lead citrate, washed with deionized distilled water (ddH₂O), and observed using TEM.

### Spearman analysis

The strength and direction of monotonic associations between *lumican* mRNA and systolic blood pressure or diastolic blood pressure were quantified using Spearman’s rank correlation coefficients, with statistical significance defined as *p < 0.05.

### Statistical analysis

Statistical analyses were performed using GraphPad Prism (version 9.3.1). Data are presented as the mean ± SEM. Normality was assessed using the Shapiro-Wilk test. For comparisons between two groups, an unpaired two-tailed Student’s t-test was used for normally distributed data; otherwise, the Mann-Whitney U test was applied. For comparisons across more than two groups, one-way ANOVA followed by Tukey’s post hoc test was used for parametric data. A *P* value < 0.05 was considered statistically significant. Image quantification was performed using ImageJ software, with the analyst blinded to the experimental groups. In all animal experiments, the pregnant dam was considered the primary statistical unit. Placental samples derived from the same dam were accordingly treated as a single, independent biological replicate (n) for inter-group comparisons to avoid pseudoreplication.

## Supplementary information

Supplementary information

## Data Availability

All data are available in the main text or the supplementary materials. The raw datasets used and/or analyzed during the current study are available from the corresponding author.
