# Supplementary material for: Lumican deficiency dysregulates trophoblast function via PI3K/AKT and P53 signaling in preeclampsia
Source: NPJ Regen Med. 2026 Jul 7;11:47. doi: 10.1038/s41536-026-00493-8 (PMC13720002; doi:10.1038/s41536-026-00493-8)
Supplement: Supplementary file 1 [file 41536_2026_493_MOESM1_ESM.pdf]

## Supplementary Figure

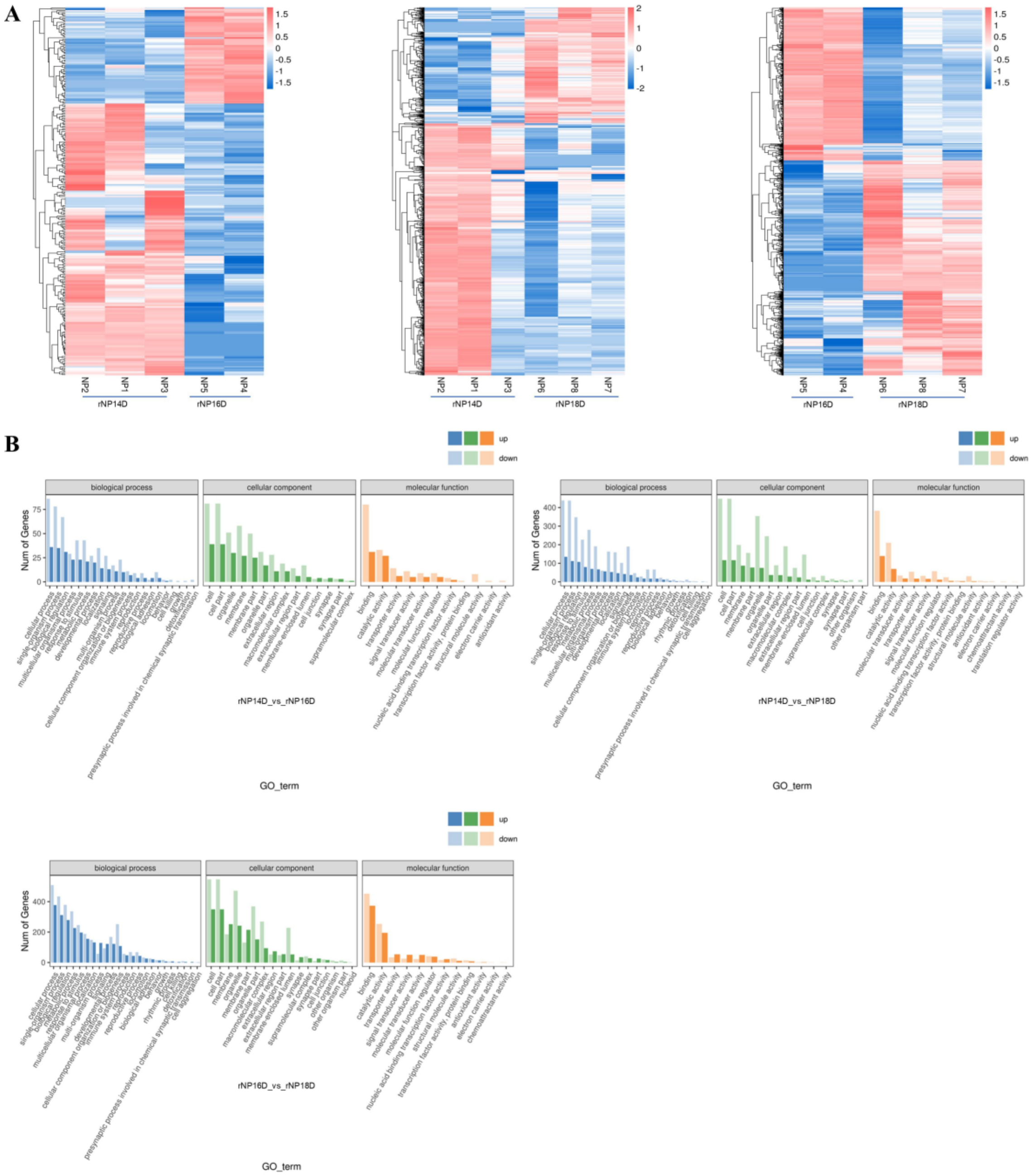


**Figure S1. Transcriptome analysis of placenta in pregnant rats.** A: Heat map; B: GO Enrichment analysis.

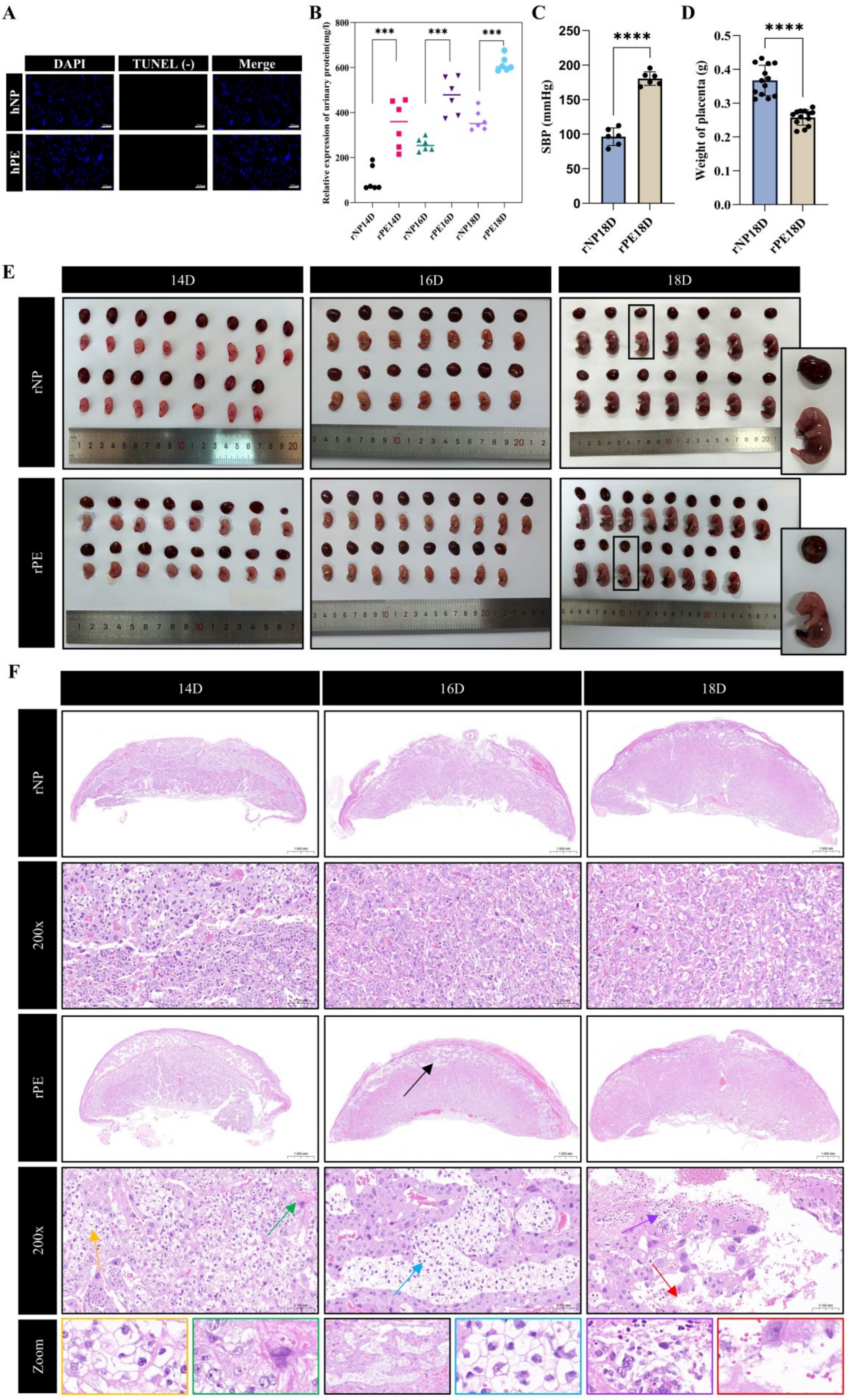

A
DAPI
TUNEL (-)
Merge
hNP
hPE
B
Relative expression of urinary protein(mg/l)
800
600
400
200
0
***
rNP14D
rPE14D
rNP16D
rPE16D
rNP18D
rPE18D
C
SBP (mmHg)
250
200
150
100
50
0
****
rNP18D
rPE18D
D
Weight of placenta (g)
0.5
0.4
0.3
0.2
0.1
0.0
****
rNP18D
rPE18D
E
14D
16D
18D
rNP
rPE
F
14D
16D
18D
rNP
200x
rPE
200x
Zoom

**Figure S2. Rat PE (rPE) model.** A: TUNEL staining negative control; B: Urinary protein content in pregnant rats, n=6; C: Systolic blood pressure (SBP) in pregnant rats, n=6; D: Placental weight; E: Placenta and fetal rats, n=13; F: H&E staining showed extensive vacuolization in the trophoblast layers (yellow arrows) and focal fibrosis (green arrows). Villi were loosely organized with edematous intervillous spaces (red arrows). The labyrinthine layer was enlarged and exhibited fibrous septa (black arrows). Focal spongiotrophoblast necrosis was replaced by fibrinous material (blue arrows). Dense inflammatory infiltrates (purple arrows) accompanied disordered and necrotic trophoblasts. Villous capillaries were significantly reduced, and findings of karyopyknosis and karyolysis indicated irreversible damage. Data are presented as the mean ± SEM. Statistical significance was determined by one-way ANOVA with Tukey's post hoc test (B) or unpaired t-test (C, D). ****$p<0.0001$, ***$p<0.001$.

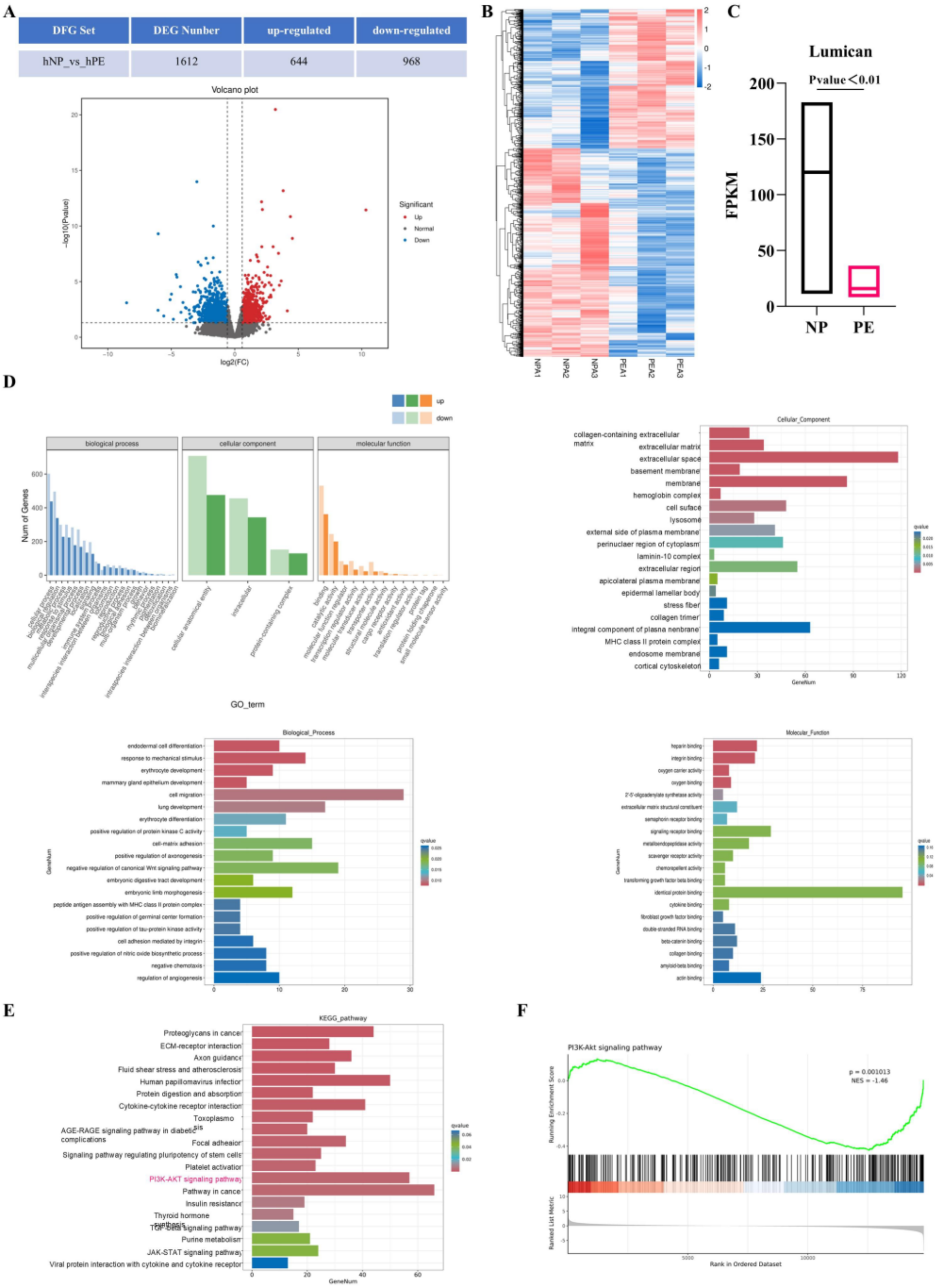


**Figure S3. Transcriptome analysis of the PI3K/AKT and P53 signaling pathway in PE placenta.** A: Volcano Plot; B: Heat map; C: FPKM value of Lumican; D: GO Enrichment; E: KEGG Enrichment; F: GSEA analysis.

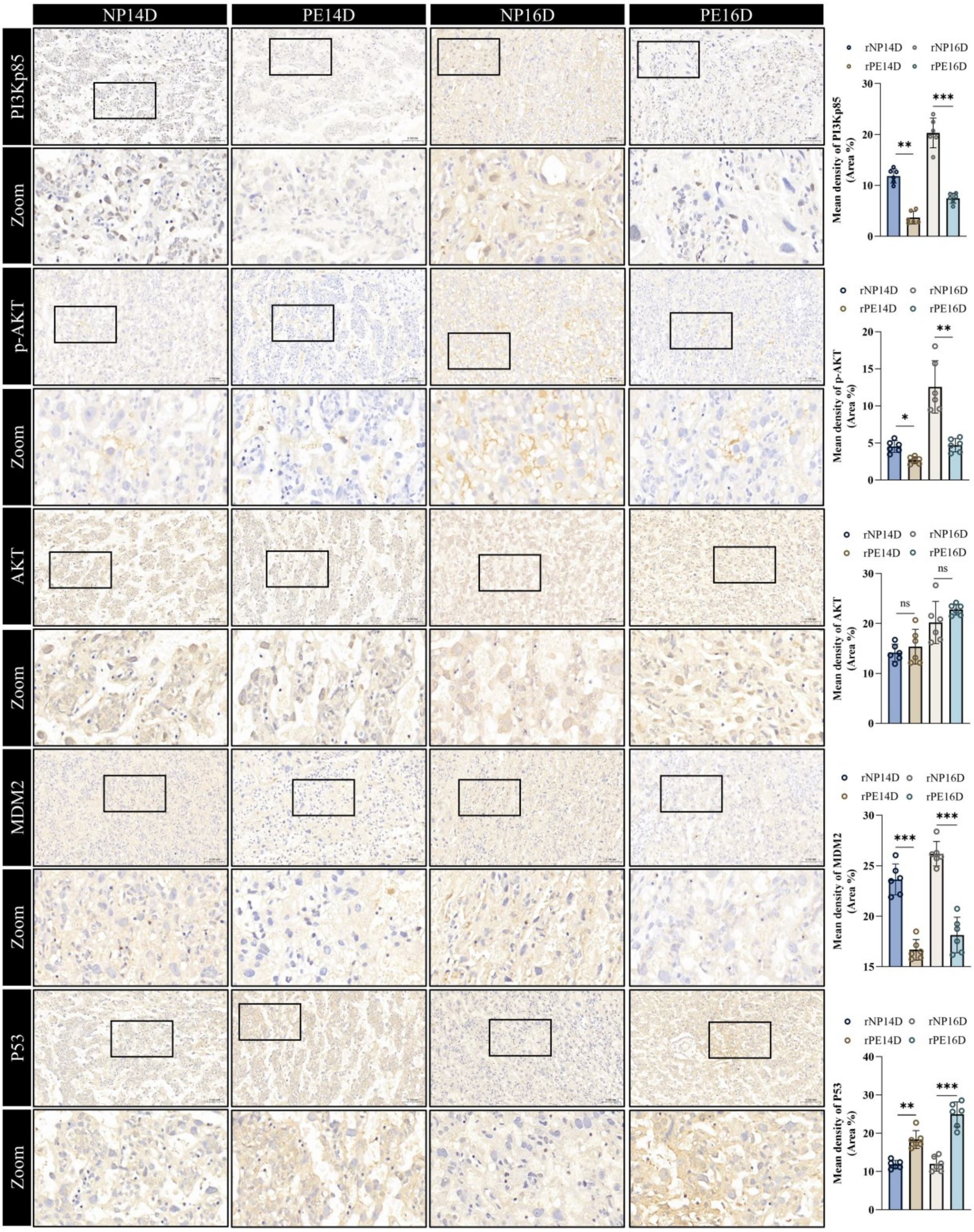


**Figure S4. Significant changes in the PI3K/AKT and P53 signaling pathway in PE placenta**. Changes in *in situ* expression levels of PI3K, AKT, MDM2, and P53 in the placentas of rats. The scale bar is 200 µm. n=6. Data are presented as the mean ± SEM. Statistical significance was determined by one-way ANOVA with Tukey's post hoc test.

*$P<0.05$, **$P<0.01$, n.s. not significant.

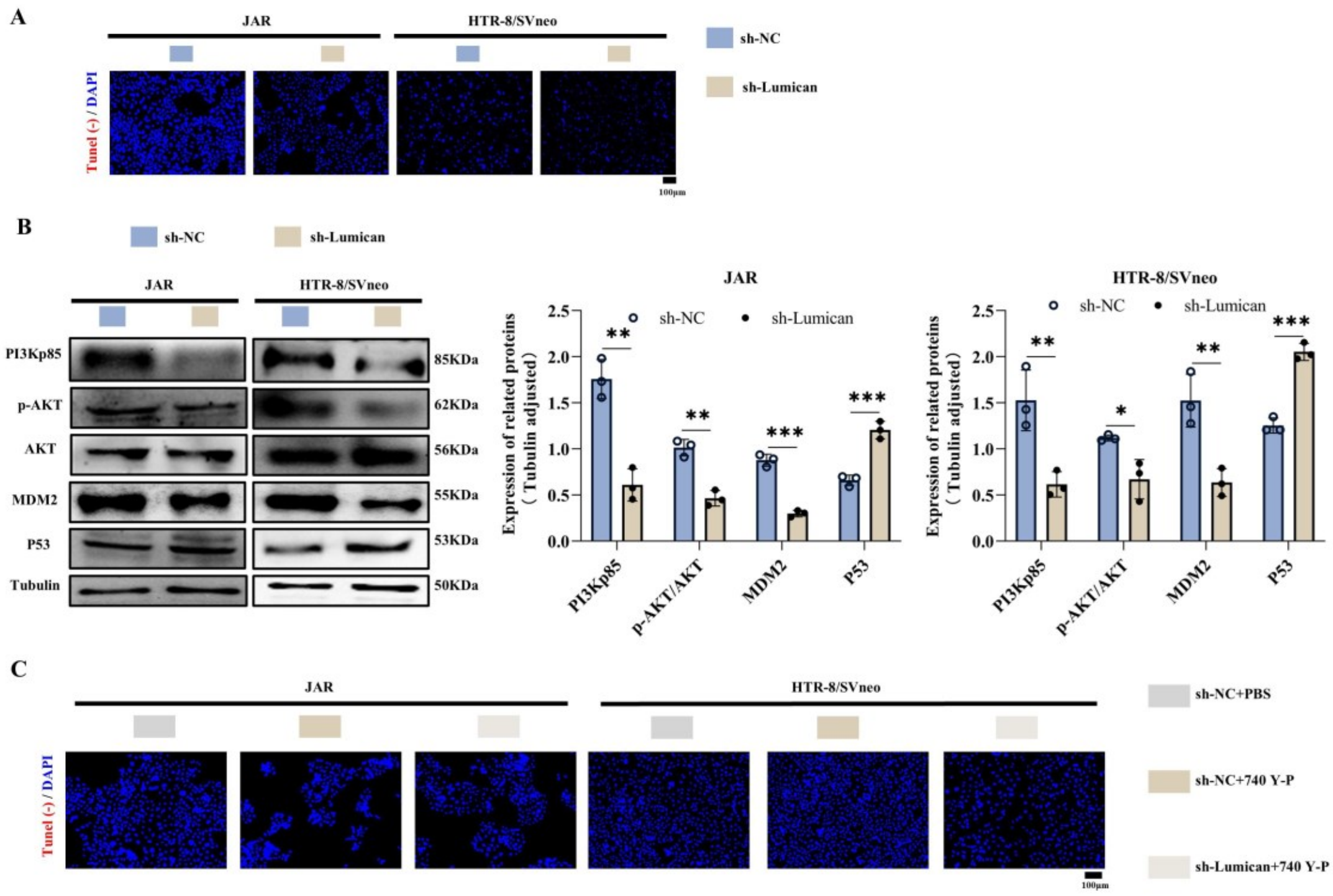


**Figure S5. Lumican downregulation regulates trophoblast cells by inhibiting PI3K / AKT and P53 signaling pathways.** A: TUNEL staining negative control; B: Changes in protein expression levels of PI3K, AKT, MDM2, and P53 in HTR-8/SVneo and JAR cells; C: TUNEL staining negative control. n=3. Data are presented as the mean ± SEM. Statistical significance was determined by unpaired t-test. ***$p$<0.001, **$p$<0.01, *$p$<0.05.

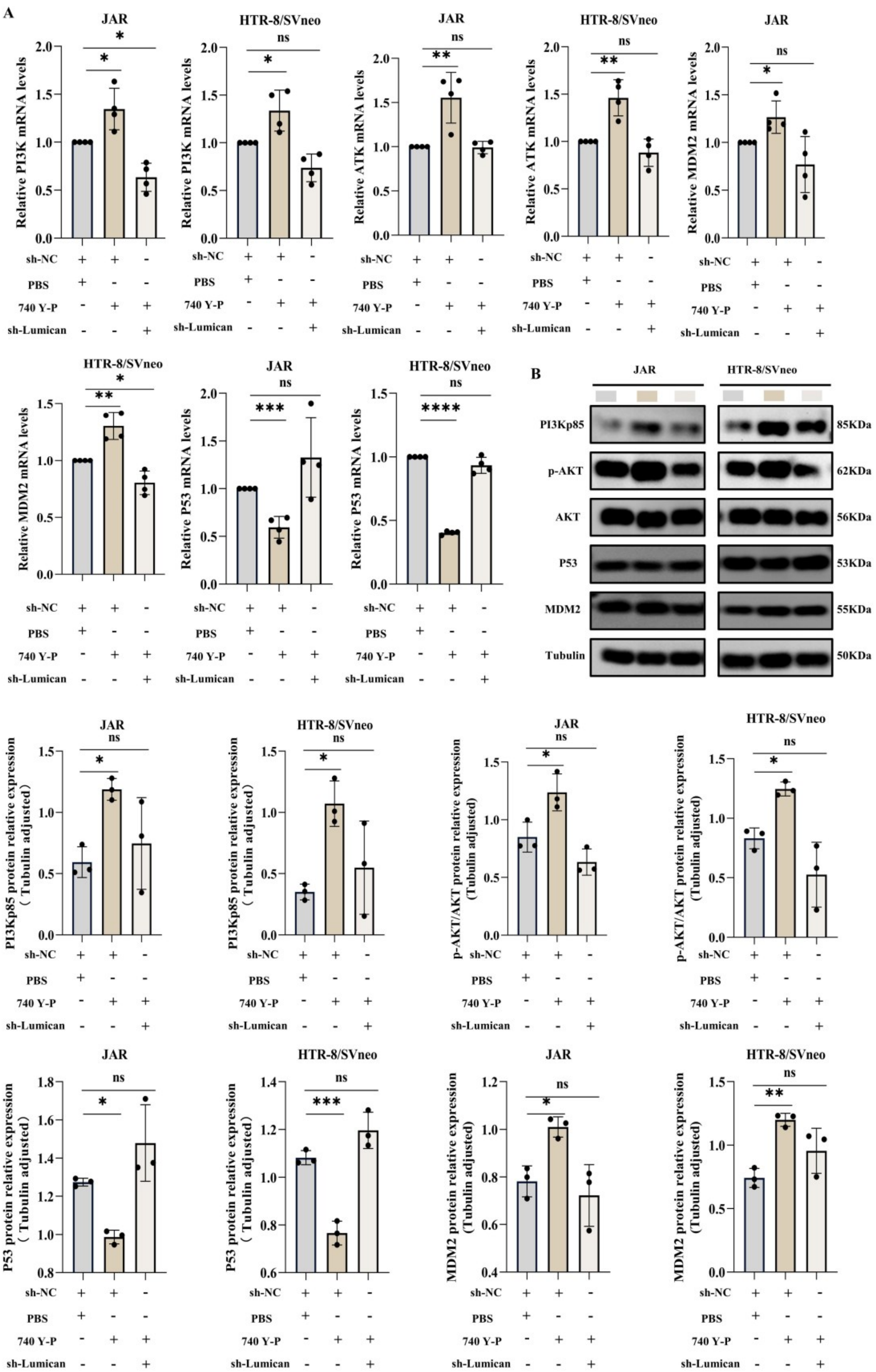


**Figure S6. Effects of *Lumican* downregulation on trophoblast function and the PI3K/AKT and P53 signaling pathway after co-treatment with 740 Y-P.** A:

mRNA expression levels of *PI3K, AKT, MDM2*, and *P53* in HTR-8/SVneo cells and JAR cells; B: Protein expression levels of PI3K, AKT, MDM2, and P53 in HTR-8/SVneo cells and JAR cells. n=3. Data are presented as the mean ± SEM. Statistical significance was determined by one-way ANOVA with Tukey's post hoc test. ****$p$<0.0001, ***$p$<0.001, **$p$<0.01, *p<0.05, n.s. not significant.

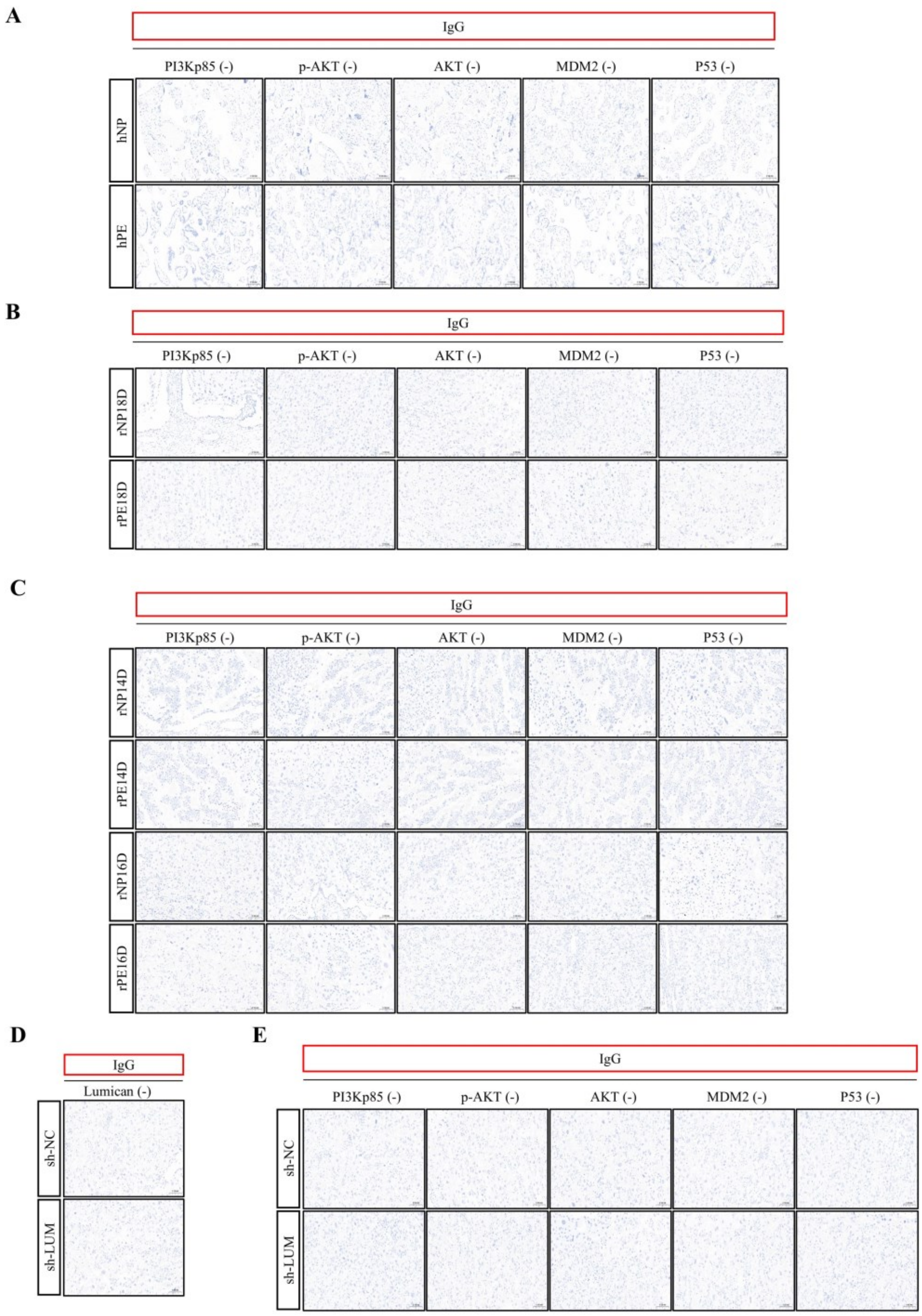


**Figure S7. Isotype control for antibody specificity.** A: *In situ* expression of PI3K, AKT, MDM2, and P53 in the human placenta was confirmed to be specific by comparison with a matched isotype control antibody; B-E: *In situ* expression of PI3K, AKT, MDM2, and P53 in the rat placenta was confirmed to be specific by comparison with a matched isotype control antibody. The scale bar is 200 μm.

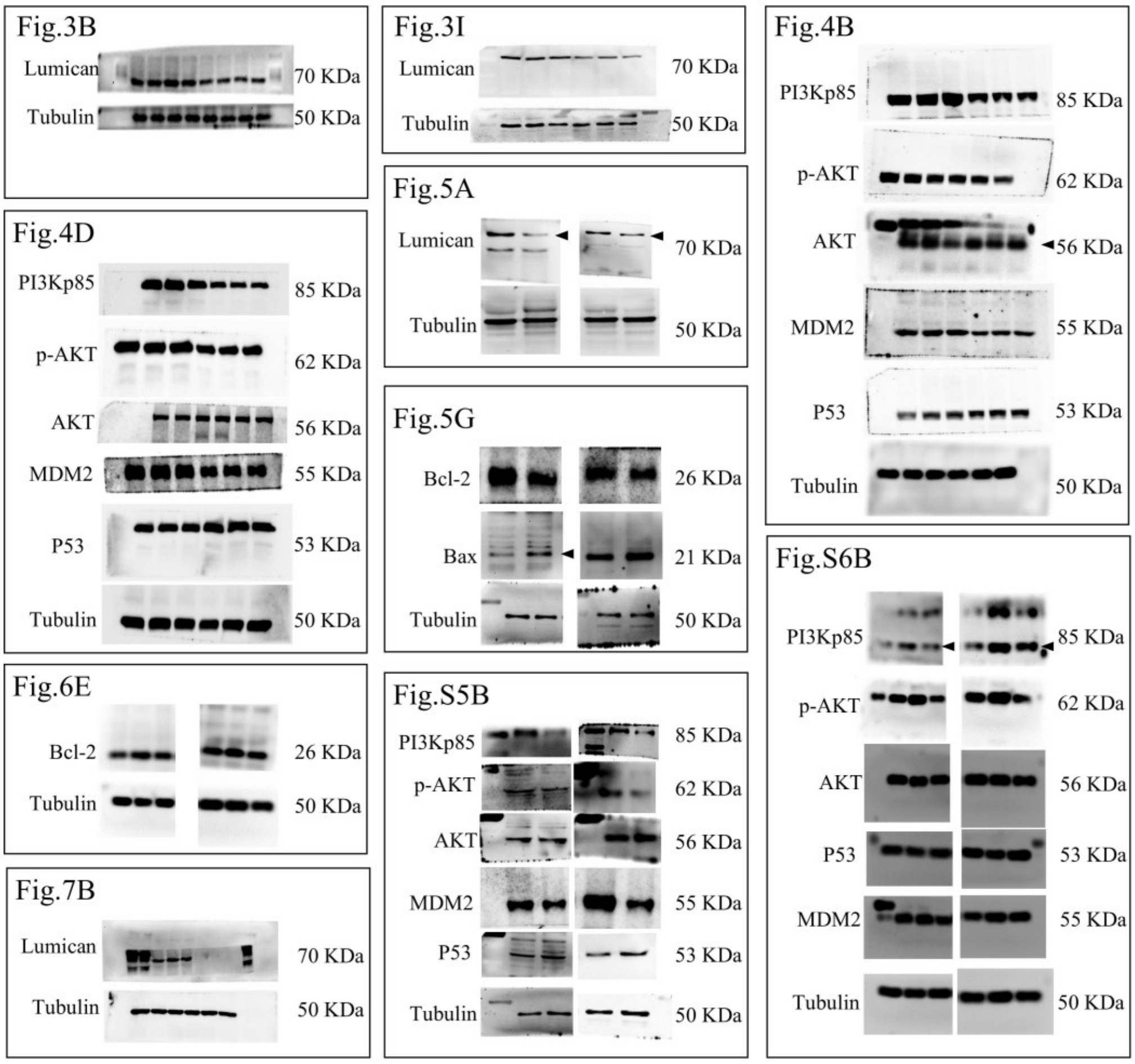


**Figure S8. Unprocessed original scans of western blots.**

**Supplementary Table**

**Table S1: Baseline characteristics of the women included in this analysis.** Abbreviations: BMI - Body mass index, SBP - Systolic blood pressure and DBP - Diastolic blood pressure. Unpaired t-test was used for comparison of means (for normally distributed data), Mann-Whitney U tests for medians (if not normally distributed), and Chi-square tests for categorical variables. 95% CI shown in square brackets for each group. 95% confidence interval (CI) shown in square brackets for each group where relevant.

| | **NP group *(n = 30)*** | **PE group (*n = 30)*** | **P-value (NP vs PE)** |
|---|---|---|---|
| **Maternal Age** (years)<br>Mean ± SEM****<br>[95%: CI] | 26.67±0.69<br>[27.76-28.05] | 31.47±0.64<br>[30.15-32.78] | <0.0001 |
| **Gestation at Delivery** (weeks)<br>Mean ± SEM****<br>[95%: CI] | 38.53±0.21<br>[38.10-38.97] | 33.93±0.31<br>[33.31-34.56] | <0.0001 |
| **BMI** (kg/m2)<br>Median (IQR) ****<br>[95%: CI] | 26.73 (23 - 33)<br>[25.86-27.60] | 31.83 (24 - 44)<br>[30.04-33.63] | <0.0001 |
| **Parity no.** (%) | | | 0.0739 |
| 0 | 14 (46.67) | 22 (73.33) | |
| 1 | 13 (43.33) | 6 (20.00) | |
| ≥ 2 | 3 (10.00) | 2 (6.67) | |
| **SBP at Delivery** (mmHg)<br>Median (IQR) ****<br>[95%: CI] | 118.4 (102 - 135)<br>[115.2-121.6] | 137.3 (122 - 159)<br>[133.4-141.2] | < 0.0001 |
| **DBP at Delivery** (mmHg)<br>Median (IQR) ****<br>[95%: CI] | 76.60 (62 - 88)<br>[74.22-78.98] | 95.77 (80 - 112)<br>[91.68-99.86] | < 0.0001 |
| **Birth weight** (g)<br>Median (IQR) ****<br>[95%: CI] | 2053 (1167 - 3520)<br>[1855-2251] | 1097 (648 - 1834)<br>[973-1220] | < 0.0001 |

**Table S2. Primer sequence**

| Gene | Accession no | Primer sequence (5' to 3') | Length |
|---|---|---|---|
| Lumican (human) | NM_002345.4 | F: CTTCAATCAGATAGCCAGACTGC | 23 |
| | | R: AGCCAGTTCGTTGTGAGATAAAC | 23 |
| Lumican (Rat) | NM_031050.2 | F: TGTCAAGAGAGTAAGGGCACAG | 22 |
| | | R: TGCACAGTTGGGTGACAGTT | 20 |
| PI3K (human) | NM_006218.4 | F: TTGATGGAGAGAGAGCAGTTCC | 22 |
| | | R: ATCTCCTCCCATGAAAATCACCT | 23 |
| PI3K (Rat) | NM_001371300.3 | F: TGGAGTGTGTGGACAGTGAAC | 21 |
| | | R: AGGTACAGATGATCCATGCGG | 21 |
| AKT (human) | NM_001014431.2 | F: ATCTCCTCCCATGAAAATCACCT | 23 |
| | | R: AAGGTGCGTTCGATGACAGT | 20 |
| AKT (Rat) | NM_033230.3 | F: TGGAGTGTGTGGACAGTGAAC | 21 |
| | | R: AGGTACAGATGATCCATGCGG | 21 |
| Bcl-2 (human) | NM_000633.3 | F: CTGGGATGCCTTTGTGGAAC | 20 |
| | | R: GGCAGGCATGTTGACTTCAC | 20 |
| Bcl-2 (Rat) | NM_016993.2 | F: GGTGAACTGGGGGAGGATTG | 20 |
| | | R: AGAGCGATGTTGTCCACCAG | 20 |
| Bax (human) | NM_001291428.2 | F: TCGAGGACGACTTCAACTATGG | 22 |
| | | R: ACAGCAAAATTAAGGCAGGACTC | 23 |
| Bax (Rat) | NM_017059.2 | F: AGACACCTGAGCTGACCTTG | 20 |
| | | R: AAGTTGCCATCAGCAAACAT | 20 |
| P53 (human) | NM_000546.6 | F: GTGACACGCTTCCCTGGATT | 20 |
| | | R: GAGCTTCATCTGGACCTGGG | 20 |
| P53 (Rat) | NM_001429993.1 | F: CCACGTGCTCACACTGGCTAA | 21 |
| | | R: ATTAGGTGACCCTGTCGCTGT | 21 |
| MDM2 (human) | NM_001145337.3 | F: AGGAGATTTGTTTGGCGTGC | 20 |
| | | R: TGAGTCCGATGATTCCTGCTG | 21 |
| MDM2 (Rat) | NM_001439517.1 | F: CTGCAGGCACCTCACAGATT | 20 |
| | | R: CCTTCAGGCTGACATCTGCT | 20 |

| | | | |
|---|---|---|---|
| β-actin (human) | NM_001101.5 | F: CATGTACGTTGCTATCCAGGC | 21 |
| | | R: CTCCTTAATGTCACGCACGAT | 21 |
| β-actin (Rat) | NM_031144.3 | F: CTGAACCCTAAGGCCAACCG | 20 |
| | | R: GACCAGAGGCATACAGGGACAA | 21 |

**Table S3. Application of antibodies**

| Antibody | Host | Isotype | Dilution rate | Manufacturer, Catalog No. |
|---|---|---|---|---|
| Lumican | Rabbit | IgG | WB: 1:1000<br>HIC: 1:200 | Proteintech, 10677-1-AP |
| PI3Kp85 | Rabbit | IgG | WB:1:2000<br>HIC: 1:200 | Proteintech, 83606-5-RR |
| AKT | Rabbit | IgG | WB:1:2000<br>HIC: 1:200 | Proteintech, 10176-2-AP |
| p-AKT | Mouse | IgG | WB:1:2000<br>HIC: 1:200 | Proteintech, 66444-1-Ig |
| Bax | Rabbit | IgG | WB:1:2000 | Proteintech, 50599-2-Ig |
| Bcl-2 | Rabbit | IgG | WB:1:1000 | Beyotime, AB2017 |
| MDM2 | Rabbit | IgG | WB:1:1000<br>HIC: 1:100 | Beyotime, AF7449 |
| P53 | Rabbit | IgG | WB:1:5000<br>HIC: 1:500 | Proteintech, 10442-1-AP |
| Tubulin | Mouse | IgG | WB:1:1000 | Beyotime, AF2835 |
| β-Actin | Mouse | IgG | WB:1:20000 | Proteintech, 66009-1-Ig |
